# Soluble and EV-bound CD27 act as antagonistic biomarkers in patients with solid tumors undergoing immunotherapy

**DOI:** 10.1186/s13046-024-03215-4

**Published:** 2024-11-08

**Authors:** Joao Gorgulho, Sven H. Loosen, Ramsha Masood, Franziska Giehren, Francesca Pagani, Gustav Buescher, Lorenz Kocheise, Vincent Joerg, Constantin Schmidt, Kornelius Schulze, Christoph Roderburg, Eva Kinkel, Britta Fritzsche, Simon Wehmeyer, Benjamin Schmidt, Paul Kachel, Christina Rolling, Julian Götze, Alina Busch, Marianne Sinn, Thais Pereira-Veiga, Harriet Wikman, Maria Geffken, Sven Peine, Urte Matschl, Markus Altfeld, Samuel Huber, Ansgar W. Lohse, Fabian Beier, Tim H. Brümmendorf, Carsten Bokemeyer, Tom Luedde, Johann von Felden

**Affiliations:** 1 Department of Oncology, Hematology and Bone Marrow Transplantation With Section of Pneumology, University Medical Centre Hamburg-Eppendorf, Hamburg, Germany; 2 University Cancer Center Hamburg – Hubertus Wald Tumorzentrum, University Medical Centre Hamburg-Eppendorf, Hamburg, Germany; 3 Department of Gastroenterology, Hepatology and Infectious Diseases, University Hospital Düsseldorf, Medical Faculty of Heinrich Heine University Düsseldorf, Düsseldorf, Germany; 4 Center for Integrated Oncology, Aachen-Bonn-Cologne-Düsseldorf (CIOABCD), Aachen, Germany; 5 I. Department of Medicine, University Medical Centre Hamburg-Eppendorf, Hamburg, Germany; 6 European Reference Network on Hepatological Diseases (ERN RARE-LIVER), Hamburg, Germany; 7 Department of Tumor Biology, University Hospital Hamburg-Eppendorf, Hamburg, Germany; 8 Institute of Transfusion Medicine, University Medical Center Hamburg-Eppendorf, Hamburg, Germany; 9 Leibniz Institute of Virology, Hamburg, Germany; 10 Department of Hematology, Oncology, Hemostaseology and Stem Cell Transplantation, Medical Faculty, RWTH Aachen University, Aachen, Germany

**Keywords:** PD-1, Liquid biopsy, Immunotherapy, Soluble immune checkpoints, Prognosis, Biomarker

## Abstract

**Background:**

The major breakthrough in cancer therapy with immune checkpoint inhibitors (ICIs) has highlighted the important role of immune checkpoints in antitumoral immunity. However, most patients do not achieve durable responses, making biomarker research in this setting essential. CD27 is a well known costimulatory molecule, however the impact of its soluble form in ICI is poorly investigated. Therefore, we aimed at testing circulating concentrations of soluble CD27 (sCD27) and CD27 bound to extracellular vesicles (EVs) as potential biomarkers to predict response and overall survival (OS) in patients undergoing ICI.

**Methods:**

Serum and plasma levels of sCD27 were assessed by immunoassay in three patient cohorts (*n* = 187) with advanced solid malignancies including longitudinal samples (*n* = 126): a training (*n* = 84, 210 specimens, *Aachen ICI*) and validation *cohort* (*n* = 70, 70 specimens, *Hamburg ICI*), both treated with ICI therapy, and a second independent validation *cohort* (*n* = 33, 33 specimens, *Hamburg non-ICI*) undergoing systemic therapy without any ICI. In a subset (*n* = 36, 36 baseline and 108 longitudinal specimens), EV-bound CD27 from serum was measured, while EV characterization studies were conducted on a fourth cohort (*n* = 45).

**Results:**

In the *Aachen* and *Hamburg ICI cohorts*, patients with lower circulating sCD27 levels before and during ICI therapy had a significantly longer progression-free survival (PFS) and OS compared to patients with higher levels, a finding that was confirmed by multivariate analysis (MVA) (*Aachen ICI:* p_PFS_ = 0.012, p_OS_ = 0.001; *Hamburg ICI:* p_PFS_ = 0.040, p_OS_ = 0.004) and after randomly splitting both cohorts into training and validation. This phenomenon was not observed in the Hamburg non-ICI *cohort*, providing a rationale for the predictive biomarker role of sCD27 in immune checkpoint blockade. Remarkably, EV-bound CD27 baseline levels and dynamics during ICI therapy also emerged as potent predictive biomarkers, acting however antagonistically to soluble sCD27, i.e. higher levels were associated with PFS and OS benefit. Combining both molecules (“multi-CD27” score) enhanced the predictive ability (HR_PFS_: 17.21 with *p* < 0.001, HR_OS_: 6.47 with *p* = 0.011).

**Conclusion:**

Soluble and EV-bound CD27 appear to have opposing immunomodulatory functions and may represent easily measurable, non-invasive prognostic markers to predict response and survival in patients undergoing ICI therapy.

**Supplementary Information:**

The online version contains supplementary material available at 10.1186/s13046-024-03215-4.

## Background

Immune checkpoint inhibitors (ICI) have revolutionized cancer therapy, being awarded Science Magazine’s breakthrough of the year 2013 [1], leading to astonishing response rates and prolonged survival across multiple cancer types and stages [2, 3]. While many patients benefit from ICI, the vast majority does not endure the desired response, making the search for biomarkers to identify suitable therapy candidates essential [4, 5]. A large number of studies have been published addressing this question, with only a very limited number of biomarkers making it into clinical routine, such as immunohistochemical PD-L1 scoring and the tumor mutational burden (TMB), with its inherent limitations e.g. the need for tissue biopsy [6–8]. Soluble isoforms of these immune checkpoints, arising from alternative splicing or proteolytic cleavage of the membrane-bound molecule, have shown promise as immunomodulators and biomarkers in different scenarios such as infections, autoimmune diseases and cancer, both within and outside the ICI setting [9–13]. Recently, the soluble isoform of CD27 has been reported to play a role in ICI therapy in renal cancer [14]. CD27 is a costimulatory immune checkpoint expressed by different immune cells, mainly T-cells. Binding to its ligand CD70, expressed mainly by antigen presenting cells (APCs), initiates a signaling cascade leading to T-cell proliferation and activation [15, 16]. This activation leads to the cleavage of CD27 by matrix metalloproteinases (MMPs) and the release of soluble sCD27 [17], which appears to act as a co-inhibitory molecule, in contrast to costimulatory membrane-bound CD27. How it ultimately unfolds this inhibitory role is still unclear, but different theories include the competitive blockade of CD70, inhibiting the initiation of costimulatory signaling through the CD27-CD70 interaction [17], or by the fact that its abundant presence is a result of chronic stimulation of the CD27-CD70 axis, revealing an exhausted T-cell phenotype [14]. Regarding its role as a predictive/prognostic biomarker, studies outside and within the cancer spectrum show its relevance, with higher levels of soluble CD27 in peripheral blood mostly being associated with worse outcome of patients [18–20]. Nevertheless, most of these studies included a small sample size, and were limited to single entities or therapeutic regimens.

In contrast to soluble biomarkers, extracellular vesicles (EVs), which are small nano-sized, membrane-embedded vesicles, have recently become of increasing interest as biomarkers for cancer (immuno-) therapy, since they are actively released from their host T-cells and function in intercellular communication, including immunomodulation [21, 22], contributing for example to ICI resistance in malignant melanoma [23]. To our knowledge, no studies have previously reported on the predictive/prognostic impact of CD27 + EVs on ICI therapy.

Based on the current evidence, we hypothesize that soluble CD27 functions as a predictive biomarker in ICI across multiple cancer types, while EV-bound CD27, despite playing an opposite immunological role, also functions as a predictive biomarker in this setting. To test this hypothesis and to gain a better mechanistic understanding of the roles of soluble CD27 and EV-bound CD27, we performed a multicenter study including 232 patients with 466 blood specimens across 12 cancer types.

## Patients and methods

### Study population

This multicenter study included four cohorts (*Aachen ICI cohort*, *Hamburg ICI cohort*, *Hamburg non-ICI cohort*, *EV characterization cohort*). Details regarding the cohorts and sampling can be found in the Supplementary Material in the section “Supplementary methods”.

### Evaluation of sCD27 serum and plasma levels

By centrifugating the whole blood samples for 10 min at 2000 g, we isolated serum and plasma samples and stored them at − 80 °C until use. Concentrations of sCD27 (serum in *Aachen ICI cohort*, plasma in *Hamburg ICI and Hamburg non-ICI cohorts*) were measured by multiplex immunoassay in accordance with the manufacturer’s instructions (Immuno-Oncology Checkpoint 14-Plex Human ProcartaPlex™ Panel 1, ThermoFisher Scientific, USA) using a Bio-Plex 200 system and Bio-Plex Manager 5.0 and 6.0 software.

### Evaluation of EV-bound CD27

**Fig. 1:**
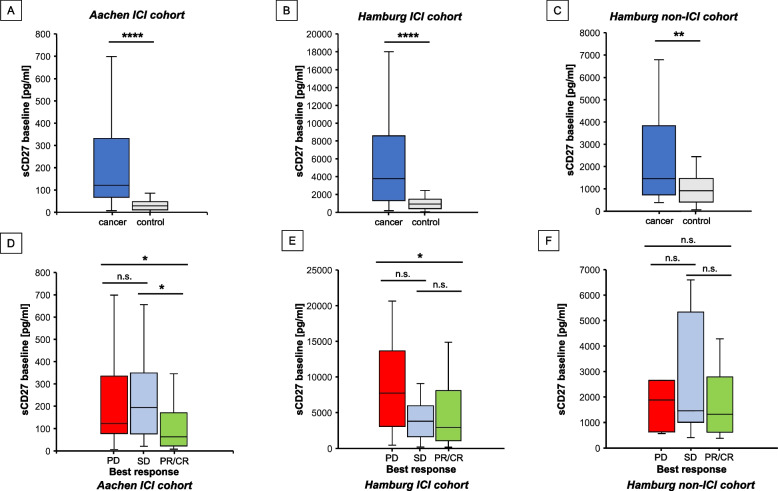
sCD27 concentrations are significantly elevated in cancer patients compared to cancer-free controls across three different cohorts and predict best response in patients undergoing immune checkpoint blockade (**A**-**C**) sCD27 concentrations in cancer patients and healthy controls: ICI treated *Aachen cohort* (*n* = 84 cancer patients, *n* = 35 healthy controls) (**A**), ICI-treated Hamburg 1 cohort (*n* = 70 cancer patients, *n* = 32 controls) (**B**), non-ICI treated Hamburg 2 cohort (*n* = 33 cancer patients, *n* = 32 controls) (**C**). (D-E) sCD27 concentrations according to best response in the ICI-treated *Aachen* (**D**) *and Hamburg ICI* (**E**) *cohorts*, as well as the *Hamburg* *non-ICI* *cohort *(**F**). *p < 0.05; **p < 0.01; ***p < 0.001, ****p < 0.0001

EV isolation was performed from cryopreserved patient serum on a subset of *n* = 45 HCC patients (*EV* *characterization* *cohort*) and of *n* = 36 HCC patients from the *Hamburg ICI cohort cohort* by differential ultracentrifugation. In detail, 1 ml of fresh serum was diluted 1:1 in filtrated PBS and filtered with 200-nm pore size filters. In a following step, serum was centrifuged at 16,000 g for 30 min, the pellet containing microparticles (MPs) was extracted and these were resuspended in filtrated PBS. Afterwards, the supernatant was twice ultracentrifuged at 100,000 g for 70 min. The pellet was again resuspended in filtrated PBS. Isolates were then stored in small aliquots at − 80 °C to avoid freezing–thawing to preserve the EV integrity and quantity. EV characterization and quality control was performed according to MISEV23 guidelines [24]. EV quantity and size was analyzed with the NanoSight NS300 (Malvern Panalytical) following the manufacturer’s instructions (Supp.Fig. 1A). Transmission electron microscopy was performed to analyze typical EV morphology (Supp.Fig. 1B). Further analyses were conducted on EV isolates. For immunolabeling, we performed ELISA in *n* = 8 patients for EV-related tetraspanins CD9, and CD63 (BIOZOL Diagnostica Vertrieb GmbH, Germany, Supp.Fig. 1C) and HCC-related marker Glypican 3 (Supp.Fig. 1D). EV-bound CD27 was analyzed using the bead-based multiplex assay panel LEGENDplex™ HU Immune Checkpoint Panel 1 Standard (BioLegend, USA) according to the manufacturer’s protocol and concentrations were normalized to total protein input.

### Statistical analysis

Following the Shapiro–Wilk Test to assess normal distribution of the data, non-parametric data was analyzed with the Mann–Whitney-U-Test and Kruskal–Wallis-H-Test. Box plot graphics demonstrate the median, quartiles and ranges. ROC curves were generated by plotting the sensitivity against 1-specificity. Kaplan–Meier curves were used to show time-dependent outcomes, such as progression-free and overall survival (PFS, OS), using the log-rank test to analyze statistical differences between subgroups. Repeated measures ANOVA compared longitudinal differences in sCD27 levels between different time points. For calculation of the ideal cut-off of soluble and EV-bound CD27 at any time point regarding PFS (to show the robustness of the PFS cut-off, this was also used for OS), we employed the “Charité cut-off finder”, a publicly available software-tool, which fits Cox proportional hazard models to the dichotomized survival status as well as the survival time and defines the optimal cut-off for the CD27 concentration with the most significant split in log-rank test [25]. For better comparability between cohorts, two ideal cut-offs were calculated: one for the absolute concentrations and the other using normalized values to the median of the respective control population. To corroborate the prognostic value of variables, uni- and multivariate Cox-regression were performed. Parameters with a *p*-value of < 0.100 in univariate testing were included in multivariate analysis. The hazard ratio (HR) and the 95% confidence interval (CI) are reported. The Pearson correlation coefficient was used for correlation analyses between soluble CD27 and EV-bound CD27. All statistical analyses were performed using SPSS 25 (SPSS, Chicago, IL, USA) and power analyses using G-Power 3.1 (Düsseldorf, Germany). A *p*-value of < 0.05 was considered statistically significant (*p < 0.05; **p < 0.01; ***p < 0.001, ****p < 0.0001).

## Results

### Study population and characteristics

#### Immunotherapy training cohort (Aachen ICI)

The *Aachen ICI training cohort* consisted of *n* = 84 predominantly male (64.2%) patients with UICC stage IV (93.8%) and III (6.2%) solid malignancies treated with ICI only, either as single (94%) or dual immunotherapy (6%) (Table 1) [12, 26–29]. The majority of patients had non-small cell lung cancer (NSCLC) (40.5%). In this cohort of heavily pretreated patients (71.5% with at least 1 prior line of therapy), the objective response rate (ORR) was 20.3%, median PFS 114 days and OS 295 days, while the median follow-up was 829 days.

**Table 1:** Characteristics of the study population

	**Immunotherapy cohort**		**Non-immunotherapy cohort**
**Parameter**	**Aachen ICI (training)**	**Hamburg ICI (validation)**	**Hamburg non-ICI (validation)**
**Cancer patients**	*n* = 84	*n* = 70	*N* = 33
Sex [%]			
male–female	64.2—35.7	77.1—22.9	39.4—60.6
Age [years, median and range]	67.5 [38–87]	69 [29–88]	65 [28–86]
BMI [kg/m^2^, median and range]	24.1 [15.9–42.3]	24.5 [15.8–40.1]	23.2 [17.6–38.9]
Tumor entity [%]			
HCC	4.7	67.1	0
NSCLC	40.5	22.9	15.2
PDAC	0	0	33.3
Melanoma	13.1	0	0
Urogenital tract	13.1	0	0
other GIT	9.7	7.1	45.5
Head and neck	10.7	0	0
Other malignancies	19.0	2.9	6.1
Staging [%]			
UICC III	6.2	0.0	12.1
UICC IV	93.8	100.0	87.3
BCLC B	0	35.6	0
BCLC C	0	64.4	0
Therapeutic agent [%]			
Immunotherapy mono	94.0	7.1	0
Immunotherapy dual	6.0	0	0
Immunotherapy + mAb (VEGF)	0	62.9	0
Immunotherapy + Chemotherapy (incl. + mAb (VEGF))	0	25.7 (30.0)	0
Chemotherapy (incl. + mAb (VEGF/EGFR))	0	0	60.1 (87.9)
TKI	0	0	12.1
Child Pugh Score			
no cirrhosis	NA	17.8	NA
A	NA	53.3	NA
B	NA	28.9	NA
ECOG PS [%]			
0–1	58.3	74.3	66.7
2	39.3	21.4	30.3
3 or more	2.3	4.3	3.0
Prior lines of systemic therapy [%]			
0	28.6	80.0	75.8
1 or more	71.5	20.0	24.2
irAE [%]			
Any	39.3	40.0	NA
G3 or higher	11.9	11.4	NA
ORR [CR/PR] [%]	20.3 [16.7/3.6]	40.0 [32.9/7.1]	35.5 [35.5/0]
PFS [days, median and 95%CI]	112 [75.17–148.83]	364 [209.61–518.39]	215 [193.05–236.95]
OS [days, median and 95%CI]	298 [58.53–537.47]	not reached	625 [79.77–1170.23]
Follow up [days, median and 95%CI]	829 [749.92–908.08]	411 [281.49–540.51]	383 [298.76–467.24]

*BMI* body mass index, *HCC* hepatocellular carcinoma, *NSCLC* non-small cell lung cancer, *GIT* gastrointestinal tract, *PDAC* pancreatic duct adenocarcinoma, *UICC* Union for International Cancer Control, *BCLC* Barcelona Clinic Liver Cancer, *ECOG PS* “Eastern Cooperative Oncology Group” performance status, *irAE* immune-related adverse effects, *ORR* objective response rate

#### Immunotherapy validation cohort (Hamburg ICI)

Regarding the *Hamburg ICI validation cohort*, it comprised *n* = 70 patients undergoing ICI either alone (7.1%) or in combination with chemotherapy (25.7%), an anti-VEGF mAb (monoclonal antibody) (62.9%) or both (4.3%) (Table 1). The tumor spectrum consisted mainly of hepatocellular carcinoma (HCC, 67.1%) and NSCLC (22.9%).

#### Chemotherapy validation cohort (Hamburg non-ICI)

A second *Hamburg non-ICI validation cohort* included *n* = 33 patients treated with chemotherapy alone (60.7%) and other agents excluding ICI (Table 1). The three main tumor entities, mostly stage IV (95.7%), were pancreatic ductal adenocarcinoma (PDAC) (33.3%), colorectal cancer (CRC) (27.3%) and NSCLC (15.2%).

#### Baseline soluble CD27 concentration is higher in cancer patients than healthy controls and associated with objective response and survival at 6 months after therapy initiation

**Fig. 2:**
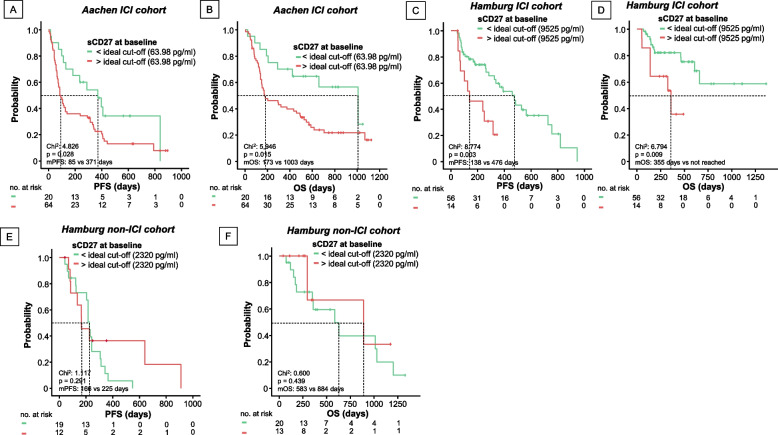
Baseline concentrations of sCD27 predict progression-free and overall survival in patients under ICI therapy. Kaplan Meier curves for PFS (**A**, **C**, **E**) and OS (**B**, **D**, **F**) stratified by ideal baseline cut-off of soluble CD27 levels calculated for PFS in the ICI-treated *Aachen cohort* (**A**-**B**) *and Hamburg 1 cohort* (**C**-**D**), as well as non-ICI treated *Hamburg 2 cohort* (**E**–**F**)

**Fig. 3:**
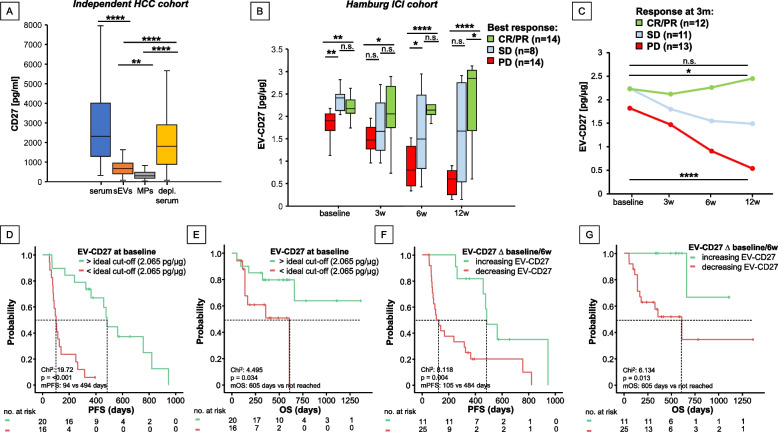
Longitudinal abundance and dynamic changes of EV-bound CD27 predict response, PFS and OS in patients undergoing ICI antagonistically to soluble CD27. **A** Levels of CD27 across serum, small extracellular vesicles (sEV), microparticles (MP), and EV-depleted serum (*n* = 45)). (**B**-**C**) EV-bound CD27 concentrations stratified by best response (**B**) and response at 3 months (**C**) over time. (**D**-**G**) Kaplan Meier curves for PFS (D, F) and OS (E, G) stratified by ideal baseline cut-off (**D**-**E**) and delta between 6 week and baseline (F-G) of EV-bound CD27 levels. *p < 0.05; **p < 0.01; ***p < 0.001, ****p < 0.0001

**Fig. 4:**
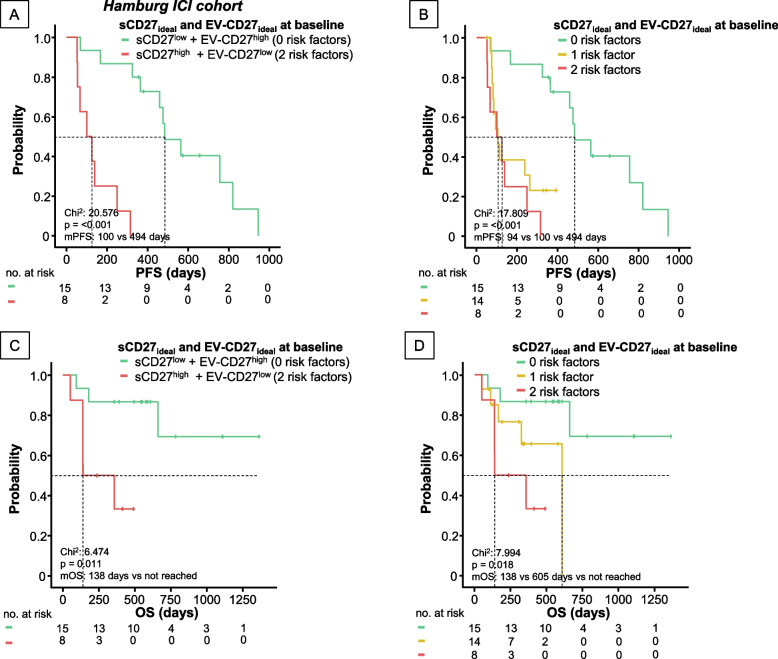
A combined immunological MCD27 score comprising sCD27 and EV-bound CD27 baseline concentrations in peripheral blood is a predictor of progression-free and overall survival in patients undergoing ICI therapy for advanced solid malignancies. Kaplan Meier curves for PFS (**A**-**B**) and OS (**C**-**D**) stratified by number of risk factors: 0 vs. 2 (**A**, **C**) and 0–2 (**B**, **D**). Risk factors are defined as either sCD27 above ideal baseline cut-off or, EV-CD27 below ideal baseline cut-off (each 1 risk factor)

As a very first step, we show that when normalizing sCD27 values to the median of the respective control population, both cohorts (*Aachen training and Hamburg-ICI validation*) show comparable ranges of sCD27 values (Supp. Figure 2A, *p* = 0.319). Then, we compared baseline soluble CD27 (sCD27) concentrations between cancer patients and healthy controls and demonstrated that these were significantly higher in cancer patients. This finding was consistent across all three cohorts (*Aachen ICI* and *Hamburg ICI cohorts*: *p* < 0.001*, Hamburg non-ICI cohort*: *p* = 0.003, Fig. 1 A-C, Supp.Fig. 2B-D) with AUC values of 0.851, 0.815, 0.709 to discriminate the two groups, respectively (Supp.Fig. 2E-G). Regarding objective and best response (OR/BR) to ICI therapy, patients in the *Aachen ICI cohort* with partial or complete response (PR/CR) had significantly lower sCD27 concentrations than patients with stable disease (SD, *p* = 0.039) and progressive disease (PD, *p* = 0.019) (Fig. 1D, Supp.Fig. 3A). This finding was confirmed in our second ICI cohort (*Hamburg ICI cohort,* *p* = 0.046 in PD vs. PR/CR patients, Fig. 1E, Supp.Fig. 3B), whereas no difference was observed in patients not receiving ICI therapy (*Hamburg non-ICI cohort*, *p* = 0.552, Fig. 1F, Supp.Fig. 3C). Baseline sCD27 predicted not only response but also survival 6 months after ICI initiation. Patients in the *Aachen ICI cohort* who had died by then had significantly higher baseline sCD27 than those who were alive (*p* = 0.005, Supp.Fig. 4A), while patients in the *Hamburg ICI cohort* show a similar, but not significant, trend (Supp.Fig. 4B). Interestingly, when patients were not treated with ICI (*Hamburg non-ICI*), deceased patients showed an opposite trend, with lower baseline levels than living patients (Supp.Fig. 4C). These data suggest sCD27 as a predictive biomarker in ICI therapy. Yet, pre-ICI sCD27 levels failed to predict irAE in the *Aachen* and *Hamburg ICI cohorts* (any irAE: p_AC_ = 0.542, p_HH1_ = 0.746; irAE ≥ G3: p_AC_ = 0.464, p_HH1_ = 0.605, Supp.Fig. 4D-E). Regarding potential confounders, we could not find significant differences in sCD27 concentrations based on tumor type (*p* = 0.137), gender (*p* = 0.22), or age (*p* = 0.62) (Supp. Figure 5).

#### Baseline concentrations of soluble CD27 predict PFS and OS following immunotherapy

Due to the significant potential of sCD27 levels in predicting response and 6-month survival in our ICI *training* and *validation* cohorts, we performed Kaplan–Meier analyses. When using the median (120.61 pg/ml) to divide patients in the *Aachen ICI cohort*, only a slight trend towards a longer PFS could be shown in patients with values below the median (*p* = 0.472, Supp.Fig. 6A), so that we calculated an absolute and relative ideal cut-off (ICO) to separate patients according to PFS (cut-off sCD27:63.98 pg/ml or 2.25 × median of controls). Patients with sCD27 levels below each cut-off had a significantly longer median PFS (mPFS) than patients above (371 vs. 85 days, *p* = 0.028, HR:1.942 [95%CI:1.059–3.561], *p* = 0.032, Fig. 2A, Supp.Fig. 6B). For OS, there was a trend towards longer OS in patients below the median (*p* = 0.083, Supp.Fig. 6C), and again a highly significant difference was observed between patients below and above the cut-off (median OS (mOS): 1003 vs 173 days, *p* = 0.015, HR: 2.366 [95%CI:1.159–4.833], *p* = 0.018, Fig. 2B, Supp.Fig. 6D). At 6 and 12 months, 85% and 70% vs. 48.4% and 40.6% of patients above and below the cut-off, respectively, were alive.

The predictive ability of sCD27 was validated in our independent ICI cohort (*Hamburg ICI cohort*, ideal cut-offs for PFS: 9525 pg/ml and 10.47 × median controls). Patients below the cut-off had a mPFS of 476 days and did not reach mOS (48.2% alive at 12 months), while patients above the cut-off had a mPFS and mOS of 138 and 355 days (14.2% alive at 12 months, p_PFS_ = 0.003, HR_PFS_:3.032 [95%CI:1.401–6.561],*p* = 0.005; p_OS_ = 0.009, HR_OS_:3.368 [95%CI:1.278–8.878],*p* = 0.014, Fig. 2C-D, Supp.Fig. 6E-F). In patients not receiving ICI (*Hamburg non-ICI cohort*), sCD27 showed no role in predicting PFS or OS, even when an ideal cut-off was calculated (ICO_HH2_:2320 pg/ml, p_PFS_ = 0.291, p_OS_ = 0.439, Fig. 2E-F). Despite the small sample size of the *Hamburg non-ICI cohort,* a post-hoc power analysis showed a sufficient power of 79% and 88% for the PFS and OS analyses, respectively, using the calculated effect size for the *Hamburg ICI cohort* (PFS d = 1.05, OS d = 1.11).

As expected, the well-established predictive biomarker tumor proportion score (TPS) of PDL-1 [30, 31] was significantly higher in patients with PR/CR as best response compared to PD in the *Aachen ICI cohort* (*n* = 51, *p* = 0.003, Supp.Fig. 7C), however, when applying the established cut-off of ≥ 1% [32], only a trend towards better PFS was seen (*p* = 0.08, Supp.Fig. 7A) and OS (Supp.Fig. 7B) was not different. In the *Hamburg ICI cohort*, neither PFS, OS or response were significantly different for TPS (*n* = 19, Supp. Figure 7D-F). Concerning neutrophile-to-lymphocyte-ratio (NLR), a often discussed biomarker for ICI [33], a significant PFS, OS and response benefit was seen in patients with a lower NLR in the *Aachen ICI cohort* (*n* = 81, p_PFS_ = 0.002, p_OS_ < 0.001, Supp.Fig. 8A-C), however, this could again not be validated in the *Hamburg ICI cohort* (*n* = 51, Supp.Fig. 8D-F). Regarding microsatellite instability, all patients with available data were stable, therefore comparative analysis was not possible. Data regarding tumor mutational burden (TMB) were only available for *n* = 3 patients, so that no analysis could be performed.

Finally, to confirm the independent role of sCD27 as a predictive biomarker for patients undergoing ICI, we performed univariate (UVA) and multivariate (MVA) analyses to exclude potential confounders, such as tumor entity, ICI regimen, ECOG and others, including the above mentioned known ICI biomarkers (Table 2, Supp.Table 1). Multivariate analyses confirmed sCD27 as an independent predictor of PFS and OS in both cohorts receiving ICI therapy (MVA *Aachen ICI cohort:* HR_PFS_:2.282 [95%CI:1.201–4.335],*p* = 0.012, HR_OS_:3.187 [95%CI:1.624–6.256],*p* = 0.001; MVA *Hamburg ICI cohort:* HR_PFS_:1.061 [95%CI:1.003–1.122],*p* = 0.040, HR_OS_:1.068 [95%CI:1.021–1.117],*p* = 0.004), while refuting it for patients not receiving ICI therapy (UVA *Hamburg non-ICI cohort*: HR_PFS_:1.050 [95%CI:0.950–1.161],*p* = 0.341, HR_OS_:0.963 [95%CI:0.772–1.200],*p* = 0.734) (Table 2). These findings further underscore the role of baseline sCD27 as a predictive biomarker in patients receiving ICI therapy across multiple cancer types. To mitigate the limitation of heterogeneity between our *Aachen ICI* and *Hamburg ICI* cohorts we performed a secondary analysis combining both cohorts to randomly split patients into new *training* and *validation cohorts,* which show homogeneity across all clinical characteristics (each *n* = 77, Supp.Table 2 for patient characteristics). Importantly, all previous results could be confirmed with this alternative approach, including the capacity of sCD27 to predict PFS and OS when applying the ideal cut-off from the new training cohort onto the new validation cohort (Supp. Figure 9–10). A new uni- and multivariate analysis corroborated the findings (Supp. Table 3). Furthermore, when proceeding towards subgroup analysis by grouping all patients of the combined cohorts into the four main tumor entities present in the manuscript (NSCLC *n* = 52, HCC *n* = 51, melanoma *n* = 11, other GI tumors *n* = 14), we could again demonstrate that higher values of soluble CD27 confer patients a worsened PFS and OS, as well as an impaired objective response, with significant differences in PFS and OS in NSCLC and HCC, OR in HCC and PFS in other GI tumors (Supp. Figure 11).

**Table 2:** Uni- and multivariate analysis training *Aachen ICI cohort* and training *Hamburg ICI cohort*

	**Aachen ICI cohort (training)**				**Hamburg ICI cohort (validation)**			
**PFS**	**univariate Cox-regression**		**multivariate Cox-regression**		**univariate Cox-regression**		**multivariate Cox-regression**	
**Parameter**	***p*-value**	**Hazard-Ratio (95% CI)**	***p*-value**	**Hazard-Ratio (95% CI)**	***p*-value**	**Hazard-Ratio (95% CI)**	***p*-value**	**Hazard-Ratio (95% CI)**
sCD27 baseline*	**0.058**	**1.906 (0.980 – 3.709)**	**0.012**	**2.282 (1.201 – 4.335)**	**0.011**	**1.048 (1.011 – 1.086)**	**0.040**	**1.061 (1.003 – 1.122)**
EV-CD27	NA	NA			**0.010**	**0.313 (0.129 – 0.761)**	**0.047**	**0.253 (0.065 – 0.985)**
Age	0.761	1.004 (0.979 – 1.030)			**0.051**	**0.970 (0.941 – 1.000)**	0.741	1.011 (0.948 – 1.078)
Sex	0.599	0.877 (0.538 – 1.430)			0.090	0.442 (0.172 – 1.135)		
UICC/BCLC stage	0.263	1.940 (0.608 – 6.193)			0.120	1.936 (0.843 – 4.449)		
Tumor entity	** < 0.001**	**1.333 (1.160 – 1.532)**	** < 0.001**	**1.361 (1.178 – 1.572)**	0.760	0.737 (1.160 – 1.250)		
Prior therapy	0.234	1.395 (0.806 – 2.416)			0.336	0.658 (0.281 – 1.543)		
ICI regimen	0.658	0.945 (0.735 – 1.214)			0.493	0.851 (0.536 – 1.350)		
ECOG PS	0.253	0.157 (0.849 – 1.861)			**0.008**	**2.300 (1.242 – 4.261)**	0.157	2.114 (0.750 – 5.954)
AFP	NA	NA			**0.051**	**1.000 (1.000 – 1.000)**	0.755	1.000 (1.000 – 1.000)
ALT	0.179	1.004 (0.998 – 1.011)			0.667	0.998 (0.990 – 1.006)		
AST	0.270	1.005 (0.996 – 1.013)			0.620	0.997 (0.985 – 1.009)		
Bilirubin	**0.101**	**1.527 (0.920 – 2.535)**	0.432	1.219 (0.744 – 2.000)	0.994	0.999 (0.840 – 1.189)		
Creatinine	0.814	1.034 (0.781 – 1.370)			0.698	0.461 (0.009 – 23.001)		
LDH	0.777	1.000 (0.998 – 1.002)			0.228	1.002 (0.999 – 1.005)		
TPS	0.109	0.993 (0.984 – 1.002)			0.224	0.970 (0.923 – 1.019)		
NLR	0.115	1.024 (0.994 – 1.055)			0.185	1.093 (0.959 – 1.246)		
**OS**	**univariate Cox-regression**		**multivariate Cox-regression**		**univariate Cox-regression**		**multivariate Cox-regression**	
**Parameter**	***p*-value**	**Hazard-Ratio (95% CI)**	***p*-value**	**Hazard-Ratio (95% CI)**	***p*-value**	**Hazard-Ratio (95% CI)**	***p*-value**	**Hazard-Ratio (95% CI)**
sCD27 baseline*	**0.008**	**2.434 (1.267 – 4.679)**	**0.001**	**3.159 (1.566 – 6.371)**	**0.012**	**1.053 (1.012 – 1.096)**	**0.004**	**1.068 (1.021 – 1.117)**
EV-CD27	NA	NA			0.398	0.582 (0.166 – 2.039)		
Age	0.658	1.006 (0.980 – 1.033)			0.547	0.987 (0.947 – 1.030)		
Sex	0.532	0.844 (0.495 – 1.438)			0.868	0.911 (0.303 – 2.737)		
UICC/BCLC stage	0.269	2.221 (0.540 – 9.136)			0.230	2.206 (0.605 – 8.043)		
Tumor entity	** < 0.001**	**1.325 (1.137 – 1.545)**	**0.002**	**1.310 (1.103 – 1.555)**	**0.100**	**0.527 (0.246 – 1.131)**	0.292	0.623 (0.258 – 1.502)
Prior therapy	0.295	1.380 (0.755 – 2.521)			**0.058**	**2.427 (0.970 – 6.070)**	0.511	1.380 (0.528 – 3.607)
ICI regimen	0.359	0.861 (0.625 – 1.185)			0.879	1.055 (0.530 – 2.098)		
ECOG PS	**0.030**	**1.593 (1.045 – 2.430)**	0.142	1.455 (0.882 – 2.401)	** < 0.001**	**4.279 (2.258 – 8.109)**	** < 0.001**	**3.894 (1.987 – 7.635)**
AFP	NA	NA			0.663	1.000 (1.000 – 1.000)		
ALT	**0.011**	**1.011 (1.002 – 1.019)**	0.050	1.009 (1.000 – 1.019)	0.351	0.993 (0.979 – 1.007)		
AST	0.049	1.010 (1.000 – 1.021)			0.409	0.982 (0.939 – 1.026)		
Bilirubin	0.146	1.512 (0.867 – 2.639)			0.471	1.074 (0.884 – 1.305)		
Creatinine	0.462	0.861 (0.578 – 1.289)			0.539	0.201 (0.001 – 33.458)		
LDH	0.868	1.000 (0.998 – 1.002)			0.879	1.000 (0.994 – 1.005)		
TPS	0.961	1.000 (0.990 – 1.009)			0.816	0.997 (0.972 – 1.022)		
NLR	**0.014**	**1.034 (1.007 – 1.063)**	**0.020**	**1.038 (1.006 – 1.070)**	0.316	1.086 (0.924 – 1.277)		

#### Soluble CD27 dynamics during immunotherapy are associated with ICI response

Interestingly, longitudinal sCD27 levels were also able to predict PFS and OS in the *Aachen ICI cohort*, both measured at an early (*n* = 72, ideal cut-off: 93.11 pg/ml, p_PFS_ = 0.062, p_OS_ = 0.009, Supp.Fig. 12A-B) and late time point during therapy (*n* = 54, ideal cut-off: 207.1 pg/ml, p_PFS_ = 0.030, p_OS_ = 0.014, Supp.Fig. 12C-D). In a further step, we investigated the dynamics of sCD27 during the three time points in the *Aachen ICI cohort*, and how these might predict patient outcome. Regarding response at the first follow-up imaging after therapy initiation (at 3 months), patients with PD had significantly higher baseline sCD27 (*p* = 0.036) compared to PR/CR. This difference was also significant for early time point sCD27 (*p* = 0.028) but not significant for the late time point sCD27 (*p* = 0.389, Supp.Fig. 13A). Repeated measures ANOVA showed no significant differences between sCD27 at the three time points for all patients with three measurements (*n* = 54, Supp.Fig. 13B). A closer look at patients who experienced a progression at the 6-month follow-up after having a partial response at 3 months, revealed substantial increases in sCD27 (*n* = 2), whereas patients with PR at 3 months who deepened remission by showing CR at 6 months (*n* = 3) showed persistently low sCD27 levels (Supp.Fig. 13C). The same analysis was performed for the 6-months follow-up, again showing a trend towards higher levels in non-responders compared to responders at all time points (Supp.Fig. 13D), and again repeated measures ANOVA analyses are not significant. Additionally, responders and patients alive at 6 months have persistently lower levels of sCD27 compared to non-responders or deceased patients (Supp.Fig. 13E). Of further note, patients who died at 6 months have significantly higher levels of sCD27 at baseline (*p* = 0.005), early (*p* = 0.07) and late time points (*p* = 0.05) compared to living patients (Supp.Fig. 13F).

We again used Kaplan–Meier analyses for PFS and OS, this time with respect to sCD27 dynamics. When using decreasing or increasing concentrations between different time points to split patients, no significant differences could be shown (Supp.Fig. 14A-D), with a relevant non-significant trend towards better OS in patients with decreasing vs. increasing sCD27 levels between baseline/late time point (*p* = 0.127, Supp.Fig. 14D). As this method may be underpowered because some patients have only minor changes in sCD27 levels and others have more pronounced changes, we decided to split patients according to the relation of their sCD27 levels and the ideal cut-offs calculated for each time point (ICO_baseline_: 63.98 pg/ml, ICO_early_: 93.11 pg/ml, ICO_late_: 207.1 pg/ml). Here, we could see relevant trends regarding PFS and significant differences regarding OS (Supp.Fig. 15A-D). Patients who stayed under the cut-off at the baseline and early time point had a longer PFS and significant longer OS than the other groups (p_PFS_ = 0.186, p_OS_ = 0.039, Supp.Fig. 15A-B). For the dynamics between baseline and late time point, an even more relevant difference was shown (p_PFS_ = 0.082, p_OS_ = 0.021, Supp.Fig. 15C-D).

In conclusion, adding to the predictive role of baseline sCD27 levels, dynamic changes can be used to monitor tumor response during ICI therapy. Particularly, sustained elevation or increase was associated with poor outcome, with the latter preceding progression in patients initially responding.

#### Longitudinal abundance and dynamic changes of membrane-bound CD27 on circulating extracellular vesicles predict objective response, PFS and OS in patients undergoing immunotherapy

To get a better insight into the differential abundance of circulating CD27, we measured CD27 expression in different peripheral blood compartments using an independent *EV characterization* *cohort* of another *n* = 45 HCC patients (all stages): whole (unprocessed) serum (median CD27 concentration: 2319.05 pg/ml), large EV fractions (i.e., microparticles) (median: 290.6 pg/ml), small EV fraction (median: 657.57 pg/ml) and EV-depleted serum (median: 1805.76 pg/ml). The intercompartmental difference was highly significant, with substantially higher levels present in serum > depleted serum > EV fractions, as expected (*p* < 0.0001, Fig. 3A), showing that the above described sCD27 comes only in a small fraction from EVs. To answer the question, whether EV-bound CD27 played the same role as sCD27, we performed further analyses. Firstly, EV characterization analysis confirmed the presence of small EVs in our isolates (Supp.Fig. 1), facilitating the investigation of the predictive biomarker capacity of membrane-bound CD27 on circulating EVs in ICI treatment. For this purpose, a subset of HCC patients from our *Hamburg ICI cohort* (*n* = 36) with *n* = 144 blood specimens at different time points before and during ICI therapy was used (baseline, 3, 6 and 12 weeks). Next, we analyzed the correlation of sCD27 concentration between patient’s plasma and EV-bound CD27 extracted from the same patient’s serum. In this case, no correlation could be found (*p* = 0.737, r_p_:-0.59, Supp.Fig. 16A). Additionally, we analyzed the concentrations of EV-CD27 from cancer-free subjects, which were significantly lower than those of HCC patients (*p* < 0.001, Supp. Figure 16B). In a further step, we analyzed the impact of EV-bound CD27 concentrations on the best response to ICI therapy, showing significant differences between PR/CR (*n* = 14) and PD (*n* = 14) patients at any given time point (p_baseline_ = 0.008, p_3w_ = 0.006, p_6w_ < 0.0001, p_12w_ < 0.0001, Fig. 3B). In contrast to the effect of sCD27, where patients with higher levels had a worse response and outcome, higher levels of EV-bound CD27 conferred a prognostic benefit. Levels of EV-bound CD27 remained relatively stable with a non-significant slight increase in responders between baseline and 12 weeks, whilst showing a significant decrease in SD (*p* = 0.021) and a dramatic decrease in PD patients (*p* < 0.0001) over time, mirroring the response to therapy (Fig. 3C). EV-bound CD27 was not only able to predict response to ICI therapy, but also PFS and OS. Again, contrarily to sCD27, patients with baseline EV-bound CD27 above a calculated ideal cut-off (2.065 pg/µg) had a better PFS (median 494 vs. 94 days, *p* < 0.0001, HR: 7.25 [95%CI: 2.655–19.81413, *p* < 0.0001, Fig. 3D) and OS (median not reached vs. 605 days, *p* = 0.034, HR: 3.57 [95%CI: 1.044–12.184], *p* = 0.042, Fig. 3E) compared to patients below the cut-off. MVA including clinical variables of prognostic relevance confirmed EV-bound CD27 as an independent predictor of PFS in the *Hamburg ICI cohort* for patients undergoing ICI (MVA: HR_PFS_: 0.253 [95%CI: 0.065–0.985], *p* = 0.047, Table 2). After 6 and 12 months, 85% and 60% of patients above and 56.3% and 31.3% below the ideal cut-off, respectively, were still alive. Not only did the static levels of EV-bound CD27 accurately predict PFS and OS, but so did their dynamics. Patients with increasing EV-bound CD27 at week 6 compared to baseline had significantly improved PFS (*p* = 0.004, HR: 7.26 [95%CI: 1.420–9.225], *p* = 0.007, Fig. 4F) and OS (*p* = HR: 8.73 [95%CI: 1.120–68.041], *p* = 0.039, Fig. 3G). This effect was also seen when considering the delta between 12 weeks and baseline (p_PFS_ = 0.004, p_OS_ = 0.219, Supp.Fig. 16C-D), but not for the delta between 3 weeks and baseline (p_PFS_ = 0.719, p_OS_ = 0.968). The accuracy of discriminating between PR/CR and PD patients using increasing/decreasing levels of EV-bound CD27 was 85.7% for ∆baseline/6 weeks and 82.1% for ∆baseline/12 weeks.

Besides soluble CD27, our findings suggest that membrane-bound CD27 on circulating EVs functions as a predictive biomarker and monitors treatment response to ICI therapy, however, with opposite outcomes compared to its soluble isoform. To better show the robustness of sCD27 measurements across different experimental settings we measured soluble sCD27 in different materials (serum and plasma) and EV-bound CD27 from the same material in different dates (March and September) and hence also different thaw-freeze cycles and multiplex plates, as well as in different materials (serum and plasma), showing comparable results with slight variations as expected (Supp. Table 4).

#### A “multi-CD27” score comprising circulating soluble and EV-bound CD27 represents a highly accurate predictor for PFS and OS in patients undergoing immunotherapy

Finally, to better reflect the complex network of immunomodulatory signaling occurring within the antitumoral response setting, we analyzed whether a composite “multi-CD27” score, using baseline soluble and EV-bound CD27 levels could predict PFS and OS better than each parameter alone. Values above the ICO for sCD27 and below the ideal cut-off for EV-bound CD27, previously reported to be prognostically adverse, were considered risk factors. Patients with 0 risk factors had a significantly improved PFS (*p* < 0.0001, HR: 17.21 [95%CI: 3.478–85.163], *p* < 0.0001, Fig. 4A-B), compared to patients with 1 or 2 risk factors. For OS, a similar highly significant prediction could be demonstrated with patients carrying 0 risk factors not reaching mOS and 80% of patients still alive 12 months after initiation of ICI therapy, whereas patients with 1 or 2 risk factors had a mOS of 605 and 138 days, respectively, with only 23.1% and 25% still alive at 12 months (*p* = 0.018, HR: 6.47 [95%CI: 1.235–33.982], *p* = 0.027, Fig. 4C-D).

## Discussion

This multi-center prospective study included 232 cancer patients and 67 healthy controls across four independent cohorts obtained from two different comprehensive cancer centers including longitudinal sampling, resulting in 533 blood samples altogether. Our results demonstrate and validate soluble CD27 as a comprehensive biomarker accurately predicting OR, PFS and OS in patients receiving ICI therapy across a wide range of malignancies. Patients with higher baseline and/or dynamic increases of sCD27 show significantly impaired response and outcome in two different ICI cohorts, whereas no difference was observed in a non-ICI cohort, demonstrating the predictive rather than prognostic capacity of our biomarker. Noteworthily, EV-bound CD27 also appears to play a potent immunomodulatory role in these patients, with opposite outcomes to its soluble isoform, with patients with lower baseline levels and/or dynamic decline over time showing significantly worse OR, PFS and OS.

Varlilumab, a CD27-agonistic mAb, has shown promise in boosting antitumor response in combination with other immunotherapeutic agents, likely due to the costimulatory effect of T cell bound CD27 [34]. However, its soluble form, sCD27, derived from proteolytic cleavage of the membrane-bound molecule, appears to play a contrasting role, mainly by competitively binding of APC-derived CD70 and inhibiting the ligand-receptor interaction of this molecule with membrane-bound CD27, thereby preventing an activation of T lymphocytes and dampening the immune response, as also suggested by our findings [17, 35, 36]. The role of sCD27 as a biomarker, a feasible and easily measurable circulating marker, has been demonstrated both within and outside the cancer spectrum. High levels of the molecule seem associated with progression of HIV infection [18] or and mirror T-cell dysfunction in autoimmune disorders [37, 38]. Studies of sCD27 related to cancer mostly depict the role of the molecule as prognostically unfavorable, with higher levels being associated with impaired OS in solid [19] and hematological malignancies [20, 39]. Regarding ICI therapy, some dichotomic data are available, with one study showing an association between high levels of sCD27 and a better outcome to ipilimumab + cancer-specific vaccine in prostatic cancer [40], while in other contexts, higher concentrations of the molecule led to worse PFS and OS for patients undergoing ICI in melanoma and clear cell renal carcinoma [14, 41]. Contrarily to our study, none of these studies has investigated the role of this soluble molecule in a pan-cancer setting, including a range of different malignancies treated with different ICI agents or including comparisons with other drug classes such as chemotherapy and VEGF antibodies. Furthermore, despite many studies depicting the relevance of immune checkpoints expressed on circulating EVs in the context of cancer (immuno)therapy [42, 43], to our knowledge no study has yet demonstrated the role of EV-bound CD27 as a predictive biomarker in patients undergoing ICI, and, more importantly, how this acts antagonistically to soluble CD27. Importantly, it is noteworthy that when measuring soluble CD27 in plasma in our ELISA-like approach, EV-bound CD27 is also detected. However, after measuring CD27 expression in different peripheral blood compartments through ultracentrifugation, we show that EV-bound CD27 only constitutes a small fraction of the measured sCD27 concentration. While the soluble molecule plays a co-inhibitory role, our findings suggest that EV-bound CD27 appears to exert a costimulatory function, potentially due to the presence of other co-factors present on the membrane that encapsulates EVs. In line with this, a recent study shows how CD27 + MPs could activate CD4 + T-cells through interaction with CD70 [44]. As mentioned before, we postulate that sCD27 competitively binds to CD70 on immune cells and prevents CD27-mediated T-cell activation, explaining why in our training (*Aachen ICI*) and validation (*Hamburg ICI*) cohorts, patients with higher levels of the circulating molecule have a worse outcome. As expected, in our non-ICI cohort (*Hamburg non-ICI*) treated with chemotherapy and other agents, which are less dependent on the host’s own immune response for antineoplastic activity, sCD27 did not seem to play a predictive role. However, this needs to interpreted with caution due to the small sample size of this cohort. Regarding the opposite immunomodulatory activity EV-bound CD27 exhibits in regards to sCD27, presenting as an immunostimulatory molecule aiding in the antitumoral response, we hypothesize that EV-bound CD27 interacts with CD70 on APCs, and with the help of its MHC complex exerts pro-immunogenic signaling in a similar fashion to CD27 expressed on T-cells or on MPs [44], aiding in the efficacy of ICI agents. In support of this thesis, studies show how, for example, extracellular vesicles carrying PD-L1 are capable of eliciting T-cell inhibition analogically to T-cell membrane bound PD-L1, since they carry MHC molecules on their surface which aid in the execution of such signaling cascades, while soluble PD-L1 is not prognostically relevant in the same setting [23, 45]. Both these antagonistic biomarkers presented by us (sCD27 and EV-bound CD27) fulfill one main task of predictive biomarkers, namely aiding in a priori decision of ideal candidates for ICI therapy before its initiation. For baseline sCD27, patients below the cut-off had a mPFS of 371 days (*Aachen ICI cohort*) and 355 days (*Hamburg ICI cohort*) compared to 85 days and 138 days for patients above. Interestingly, other biomarkers such as TPS and NLR [30] could be confirmed in the *Aachen ICI cohort*, but not in the* Hamburg ICI cohort*, likely due to a small sample size of patients with available data. For EV-bound CD27, patients below the baseline ICO were at 7.3 times higher risk of progression and 3.6 times higher risk of death. For the composite “multi-CD27 score”, 80% of patients with 0 risk factors were alive at 12 months, while for 1 or 2 risk factors, only about 25% still lived by then.

Radiological findings continue to play an unequivocal role in steering oncological therapies, carrying, however, numerous limitations ranging from availability and expenses to accuracy of differentiating between responders and non-responders, unable to, in some cases, discern viable tumor tissue from necroses or, in the era of immunotherapy, accurately identify pseudo progression [46, 47]. These limitations convey biomarkers a further fundamental task, namely aiding physicians in the a posteriori monitoring during therapy, which is also fulfilled by soluble and EV-bound CD27. In the *Aachen ICI cohort*, patients who stayed below the ideal cut-off between baseline and early time point had a significantly improved mPFS of 396 days, while patients who crossed or stayed above it had a mOS of 130 and 106 days, respectively. Importantly, assessing these dynamics at a median of 4 weeks after therapy initiation represent a much earlier time point than an initial radiological study performed to surveil the impact of therapy. Similarly, when assessing early EV-bound CD27 dynamics, PR/CR and PD patients at a 3-months radiological follow-up, could be accurately identified already at the 6-weeks sampling in 85.7% of cases, again preceding assessment via imaging by 6 weeks.

Despite the exciting results, some limitations are noteworthy. First, our cohorts present heterogenous composition. While age, sex, and BMI were comparable, tumor entities and ICI agents differed. Second, the non-ICI cohort (*Hamburg non-ICI*) has a relatively small sample size (showing however sufficient power) and differed from the others in terms of sex and tumor entities (mainly GIT and PDAC), which is expected based on approved therapies for these tumor types. Further multicenter prospective studies are warranted to confirm the predictive capacity of sCD27 as a biomarker in ICI-based therapies, but not in non-ICI therapies. Another important limitation is the fact that the range of values for absolute sCD27 concentration is quite different between the *training* and *validation* cohorts. To further investigate this phenomenon, we measured soluble CD27 and EV-bound CD27 in different materials (serum and plasma) from five patients from the Hamburg cohort, showing comparable results for EV-bound CD27 and differences for soluble CD27 (Supp. Table 4). However, these differences had a significantly smaller magnitude compared to differences between *Aachen* and *Hamburg* cohorts, suggesting that the differences between cohorts are rather caused by local preanalytical aspects (different freeze thaw-cycles, sample processing and multiplex software (Bioplex 5.0 in Aachen vs Bioplex 6.0 in Hamburg)). However, for other cytokines a material-dependent difference has been reported [48], which is likely present in our data as well, accounting for the remaining, smaller differences regarding sCD27 displayed in Supp. Table 4. To overcome both limitations, we present on the one hand normalized ideal values and cut-offs for sCD27 (based on the ratio to the median of controls), showing comparable ranges between cohorts and sample type, underscoring the biological robustness of our data (Supp. Figure 2A). On the other hand, more importantly, sCD27 findings could be confirmed when randomly splitting ICI cohorts into new *mixed training* and *validation* cohorts, which show homogeneity across a wide range of clinical parameters such as tumor entities and delivered therapeutic agents. The difference in the range of the absolute concentrations does not, to our mind, in any way disprove the fact that sCD27 is a highly relevant predictive biomarker in patients undergoing ICI therapy, but may highlight the importance of homogeneity in sampling and storage in case of a future establishment of the molecule as a biomarker in clinical practice.

## Conclusion

Despite the need for higher-volume confirmatory multicenter approaches, our findings demonstrate and validate how soluble CD27 has a clear role as a predictive biomarker for patients before and during immune checkpoint blockade, while EV-bound CD27 acts in an antagonistic fashion, possibly by mediating an antitumoral immune response boost through MHC-derived costimulatory signaling. Both molecules represent blood-based liquid biopsy approaches and are readily measurable and accessible predictive biomarkers in this setting. Combining the two into a promising "multi-CD27 score" will provide a better representation of the highly complex cancer-patient network, allowing the development of a highly relevant biomarker for ICI therapy.

## Supplementary Information

Supplementary Material 1.

Supplementary Material 2.

## Data Availability

Data will be made available, upon reasonable request, by the corresponding author.

## Abbreviations

ICI: Immune checkpoint inhibitors
EV: Extracellular vesicles
OS: Overall survival
PFS: Progression-free survival
sCD27: Soluble CD27
TMB: Tumor mutational burden
APC: Antigen presenting cell
MMP: Matrix metalloproteinase
AC: Aachen
HH: Hamburg
PD: Progressive disease
PR: Partial remission
SD: Stable disease
CR: Complete remission
GI: Gastrointestinal
NSCLC: Non-small cell lung cancer
SCLC: Small cell lung cancer
mAb: Monoclonal antibody
PDAC: Pancreatic ductal adenocarcinoma
CRC: Colorectal cancer
OR: Objective response
BR: Best response
irAE: Immune-related adverse events
MP: Microparticle
