# Supplementary material for: Soluble and EV-bound CD27 act as antagonistic biomarkers in patients with solid tumors undergoing immunotherapy
Source: J Exp Clin Cancer Res. 2024 Nov 8;43:298. doi: 10.1186/s13046-024-03215-4 (PMC11545160; doi:10.1186/s13046-024-03215-4)
Supplement: Supplementary file 1 — Supplementary Material 1. [file 13046_2024_3215_MOESM1_ESM.docx]

**Supplementary Material**

**Soluble and EV-bound CD27 act as antagonistic biomarkers in patients with solid tumors undergoing immunotherapy**

Joao Gorgulho^1,2*^, Sven H. Loosen^3,4,*^, Ramsha Masood^5^, Franziska Giehren^5^, Francesca Pagani^5^, Gustav Buescher^5^, Lorenz Kocheise^5^, Vincent Joerg^5^, Constantin Schmidt^5^, Kornelius Schulze^5^, Christoph Roderburg^2,3^, Eva Kinkel^2^, Britta Fritzsche^2^, Simon Wehmeyer^1^, Benjamin Schmidt^1^, Paul Kachel^1^, Christina Rolling^1^, Julian Götze^1^, Alina Busch^1^, Marianne Sinn^1^, Thais Pereira^6^, Harriet Wikman H^6^, Maria Geffken^7^, Sven Peine^7^, Urte Matschl^8^, Markus Altfeld^8^, Samuel Huber^5^, Ansgar W. Lohse^5^, Fabian Beier^4,9^, Tim H. Brümmendorf^4,9^, Carsten Bokemeyer^1,2^, Tom Luedde^3,4,#^, Johann von Felden^5,#^

^1^ Department of Oncology, Hematology and Bone Marrow Transplantation with Section of Pneumology, University Medical Centre Hamburg-Eppendorf, Martinistraße 52, 20251 Hamburg, Germany.

^2^ University Cancer Center Hamburg – Hubertus Wald Tumorzentrum, University Medical Centre Hamburg-Eppendorf, Martinistraße 52, 20251 Hamburg

^3^ Department of Gastroenterology, Hepatology and Infectious Diseases, University Hospital Düsseldorf, Medical Faculty of Heinrich Heine University Düsseldorf, 40225 Düsseldorf, Germany

^4^ Center for Integrated Oncology Aachen-Bonn-Cologne-Düsseldorf (CIOABCD), Aachen, Germany

^5^ I. Department of Medicine, University Medical Centre Hamburg-Eppendorf, Martinistraße 52, 20251 Hamburg, Germany

^6^ Department of Tumor Biology, University Hospital Hamburg-Eppendorf, 20246 Hamburg, Germany.

^7^ Institute of Transfusion Medicine, University Medical Center Hamburg-Eppendorf, 20246 Hamburg, Germany

^8^ Leibniz Institute of Virology, Hamburg, Germany

^9^ Department of Hematology, Oncology, Hemostaseology and Stem Cell Transplantation, Medical Faculty, RWTH Aachen University, Aachen, Germany

* These authors share main authorship

^#^ These authors share last authorship

**Table of Contents**

Supplementary Methods (page 3)

Supplementary Tables (page 4-8):

- Supplementary Table 1 (page 4)

- Supplementary Table 2 (page 5)

- Supplementary Table 3 (page 6)

- Supplementary Table 4 (page 7)

Supplementary Figures (page 8-27)

- Supplementary Figure legends (page 8-11)

- Supplementary Figure 1 (page 12)

- Supplementary Figure 2 (page 13)

- Supplementary Figure 3 (page 14)

- Supplementary Figure 4 (page 15)

- Supplementary Figure 5 (page 16)

- Supplementary Figure 6 (page 17)

- Supplementary Figure 7 (page 18)

- Supplementary Figure 8 (page 19)

- Supplementary Figure 9 (page 20)

- Supplementary Figure 10 (page 21)

- Supplementary Figure 11 (page 22)

- Supplementary Figure 12 (page 23)

- Supplementary Figure 13 (page 24)

- Supplementary Figure 14 (page 25)

- Supplementary Figure 15 (page 26)

- Supplementary Figure 16 (page 27)

## **Supplementary methods**

**Study population**

As a first step, we focused on a *training cohort* of n=84 patients with advanced-stage solid malignancies treated with an ICI, prospectively recruited from August 2017 to September 2019 at the oncological outpatient clinic at the University Hospital RWTH Aachen (*Aachen ICI cohort*, Table 1) (12, 24-28). Next, we aimed to validate the results of the *Aachen ICI cohort* using two independent cohorts *(Hamburg ICI and Hamburg non-ICI). Hamburg ICI* was prospectively recruited from February 2020 to October 2023, whereas *Hamburg non-ICI* was recruited from March 2016 to September 2023, both at the University Medical Centre Hamburg-Eppendorf. Both cohorts included patients with advanced solid malignancies, with patients in the *Hamburg ICI cohort* (n=70) being treated with an ICI-containing regimen, in some cases combined with chemotherapy or other agents, whereas patients in the *Hamburg non-ICI cohort* (n=33) were treated with any systemic regimen, explicitly not including an ICI (Table 1). In all three cohorts*,* blood samples were collected prior to therapy. Additionally, sequential blood specimens were collected in subsets of *Aachen ICI* (early time point n=72: after 2-3 cycles, late time point n=54: after 4-6 cycles) and *Hamburg ICI* (after 3, 6 and 12 weeks of therapy, each n=36). Additionally, blood was drawn from two control populations at each center (n=32 for *Aachen cohort*, n=35 for *Hamburg cohorts)*, composed of cancer-free blood donors with age ranging from 18-65 years. EV characterization experiments were conducted on an independent cohort of 45 HCC patients from Hamburg containing patients across different stages of the disease and treatment regimens. Clinical data was obtained from medical chart reviews, incl. radiologic response assessment according to RECIST (performed by trained radiologist) and immunohistochemical scores (performed by trained pathologist).

## **Supplementary tables**

|  | **Immunotherapy cohorts** | | **Non-immunotherapy cohort** |
| --- | --- | --- | --- |
| **Parameter** | **Aachen ICI (training)** | **Hamburg ICI (validation)** | **Hamburg non-ICI (validation)** |
| sCD27 baseline [pg/ml] | 120.61 [7.18-2526.50] | 3767.92 [175.25-42229.74] | 1454.92 [382.96-22023.51] |
| sCD27 early time point [pg/ml] | 140.66 [2.02-3986.43] | NA | NA |
| sCD27 late time point [pg/ml] | 135.90 [5.63-1291.16] | NA | NA |
| EV-CD27 baseline [pg/µg] | NA | 2.10 [1.03-3.01] | NA |
| EV-CD27 12 weeks [pg/µg] | NA | 0.81 [0.15-3.13] | NA |
| Haemoglobin [g/l] | 11.95 [7.6-17.60] | 12.95 [8.9-17.3] | 12.00 [8.8-15.5] |
| Platelets [cells/nl] | 243.0 [112.0-693.0] | 224.5 [62.0-796.0] | 256.0 [120.0-618.0] |
| Leucocytes [cells/nl] | 7.1 [3.1-29.1] | 7.85 [2.0-36.0] | 6.7 [2.8-19.4] |
| Neutrophils [cells/nl] | 4.9 [1.7-26.0] | 5.0 [1.8-26.8] | NA |
| Lymphocytes [cells/nl] | 1.0 [0.3-6.4] | 1.2 [0.2-3.2] | NA |
| NLR | 4.7 [0.43-44.5] | 4.1 [1.6-19.8] | NA |
| Bilirubin [mg/dl] | 0.35 [0.10-3.7] | 0.6 [0.2-11.9] | 0.4 [0.2-7.1] |
| AST [U/l] | 25.0 [10.0-187.0] | 28.0 [13.0-327.0] | 27.0 [10.0-152.0] |
| ALT [U/l] | 18.0 [7.0-179.0] | 34.5 [9.0-279.0] | 20.0 [9.0-363.0] |
| LDH [U/l] | 219.0 [8.2-1273.0] | 248.0 [169.0-851.0] | 201.0 [124.0-2497.0] |
| Creatinine [mg/dl] | 0.87 [0.37-6.09] | 0.83 [0.42-1.50] | 0.84 [0.49-1.87] |
| AFP [µg/l] | NA | 51.8 [1.7-29106.6] | NA |
| CA19-9 [kU/l] | NA | 745.9 [22.1-1449.2] | 1307.0 [96.70-162962.3] |
| NSE [µg/l] | NA | 373.0 [143.0-7888.40] | 384.0 [384.0-384.0] |
| TPS [%] | 5.0 [0.0-100.0] | 4.0 [0.0-100.0] | NA |

**Supplementary Table 1.** Overview laboratorial parameters training and validation cohort

AFP: alpha fetoprotein, ALT: alanine transaminase, AST: aspartate transaminase, CA19-9: carbohydrate antigen 19-9, LDH: lactate dehydrogenase, NSE: neuron-specific enolase, TPS: tumor proportion score (PDL-1 in %), NLR: neutrophil lymphocyte ratio

**Supplementary Table 2.** Characteristics of mixed training and validation cohorts

| **Parameter** | **Mixed training** | **Mixed validation** |
| --- | --- | --- |
| **Cancer patients** | n=77 | n=77 |
| Initial cohort |  |  |
| Aachen ICI | 53.2 | 55.8 |
| Hamburg ICI | 46.8 | 44.2 |
| Sex [%] |  |  |
| male-female | 59.7 – 40.3 | 80.5 – 19.5 |
| Age [years, median and range] | 65.5 [29-88] | 69.0 [51-87] |
| BMI [kg/m^2^, median and range] | 24.1 [15.8-40.1] | 24.3 [15.9-42.3] |
| Tumor entity [%] |  |  |
| HCC | 29.9 | 36.4 |
| NSCLC | 29.9 | 35.1 |
| Melanoma | 6.5 | 7.8 |
| Urogenital tract | 9.1 | 5.2 |
| other GIT | 13.0 | 5.2 |
| Head and neck | 3.9 | 7.8 |
| Other malignancies | 7.8 | 2.6 |
| Staging [%] |  |  |
| UICC III | 9.1 | 0.0 |
| UICC IV | 90.9 | 100.0 |
| BCLC B | 42.1 | 30.8 |
| BCLC C | 57.9 | 69.2 |
| Therapeutic agent [%] |  |  |
| Immunotherapy mono | 53.2 | 54.5 |
| Immunotherapy dual | 2.6 | 5.2 |
| Immunotherapy + mAb (VEGF) | 24.7 | 32.5 |
| Immunotherapy + Chemotherapy (incl. + mAb (VEGF)) | 16.9 (19.5) | 6.5 (7.8) |
| Child Pugh Score |  |  |
| no cirrhosis | 31.6 | 7.7 |
| A | 47.4 | 57.7 |
| B | 21.1 | 34.6 |
| ECOG PS [%] |  |  |
| 0-1 | 67.5 | 63.6 |
| 2 | 29.9 | 33.8 |
| 3 or more | 2.6 | 2.6 |
| Prior lines of systemic therapy [%] |  |  |
| 0 | 51.9 | 51.9 |
| 1 or more | 48.1 | 48.1 |
| ORR [%] | 27.3 | 31.2 |
| PFS [days, median and 95%CI] | 283.0 [135.9-430.1] | 262.0 [101.7-422.3] |
| OS [days, median and 95%CI] | 494.0 [305.5-682.5] | 615.0 [447.6-782.4] |
| Follow up [days, median and 95%CI] | 542.0 [483.8-600.2] | 623.0 [393.9-852.1] |

BMI: body mass index, ECOG PS: “Eastern Cooperative Oncology Group” performance status, NSCLC: non-small cell lung cancer, GIT: gastrointestinal tract, CTC: common toxicity criteria

|  | **Mixed training cohort (n=77)** | | | | **Mixed validation cohort (n=77)** | | | |  |  |  |  |
| --- | --- | --- | --- | --- | --- | --- | --- | --- | --- | --- | --- | --- |
| **PFS** | **univariate Cox-regression** | | **multivariate Cox-regression** | | **univariate Cox-regression** | | **multivariate Cox-regression** | |  |  |  |  |
| **Parameter** | **p-value** | **Hazard-Ratio (95% CI)** | **p-value** | **Hazard-Ratio (95% CI)** | **p-value** | **Hazard-Ratio (95% CI)** | **p-value** | **Hazard-Ratio (95% CI)** |  |  |  |  |
| sCD27 baseline* | **0.043** | **1.023 (1.001 – 1.046)** | **0.029** | **1.027 (1.003 – 1.051)** | **0.028** | **1.026 (1.003 – 1.050)** | **0.036** | **1.038 (1.002 – 1.074)** |  |  |  |  |
| Age | 0.885 | 0.998 (0.975 – 1.022) |  |  | 0.160 | 0.978 (0.947 – 1.009) |  |  |  |  |  |  |
| Sex | 0.606 | 0.861 (0.486 – 1.523) |  |  | 0.859 | 1.060 (0.557 – 2.020) |  |  |  |  |  |  |
| UICC/BCLC stage | 0.299 | 1.869 (0.574 – 6.090) |  |  | 0.069 | 2.847 (0.922 – 8.795) |  |  |  |  |  |  |
| Tumor entity | 0.260 | 1.082 (0.943 – 1.240) |  |  | **0.009** | **1.255 (1.059 – 1.488)** | **0.012** | **1.523 (1.099 – 2.111)** |  |  |  |  |
| Prior therapy | **0.066** | **1.683 (0.965 – 2.933)** | 0.271 | 1.386 (0.775 – 2.477) | 0.285 | 1.339 (0.784 – 2.287) |  |  |  |  |  |  |
| ICI regimen | **0.048** | **0.748 (0.561 – 0.997)** | 0.156 | 0.810 (0.606 – 1.083) | 0.293 | 0.881 (0.695 – 1.116) |  |  |  |  |  |  |
| ECOG PS | **0.085** | **1.576 (0.938 – 2.646)** | 0.098 | 1.573 (0.920 – 2.689) | **0.038** | **1.704 (1.029 – 2.821)** | 0.154 | 1.887 (0.788 – 4.520) |  |  |  |  |
| AFP | 0.162 | 1.000 (1.000 – 1.000) |  |  | 0.142 | 1.000 (1.000 – 1.000) |  |  |  |  |  |  |
| ALT | 0.379 | 0.997 (0.990 – 1.004) |  |  | 0.546 | 1.002 (0.995 – 1.010) |  |  |  |  |  |  |
| AST | 0.606 | 0.998 (0.991 – 1.005) |  |  | 0.944 | 1.000 (0.987 – 1.014) |  |  |  |  |  |  |
| Bilirubin | 0.902 | 1.013 (0.819 – 1.255) |  |  | 0.526 | 0.925 (0.726 – 1.177) |  |  |  |  |  |  |
| Creatinine | 0.464 | 1.242 (0.695 – 2.221) |  |  | 0.899 | 1.022 (0.727 – 1.438) |  |  |  |  |  |  |
| LDH | 0.510 | 1.001 (0.998 – 1.003) |  |  | 0.549 | 0.999 (0.996 – 1.002) |  |  |  |  |  |  |
| TPS | 0.664 | 0.997 (0.985 – 1.010) |  |  | **0.060** | **0.988 (0.976 – 1.001)** | **0.036** | **0.984 (0.969 – 0.999)** |  |  |  |  |
| NLR | 0.132 | 1.066 (0.981 – 1.158) |  |  | **0.065** | **1.030 (0.998 – 1.064)** | 0.328 | 1.020 (0.981 – 1.061) |  |  |  |  |
| **OS** | **univariate Cox-regression** | | **multivariate Cox-regression** | |  | |  | |  | 1.093 (0.959 – 1.246) | 0.328 | 1.020 (0.981 – 1.061) |
| **Parameter** | **p-value** | **Hazard-Ratio (95% CI)** | **p-value** | **Hazard-Ratio (95% CI)** | **p-value** | **Hazard-Ratio (95% CI)** | **p-value** | **Hazard-Ratio (95% CI)** |  |  |  |  |
| sCD27 baseline* | **0.003** | **1.035 (1.011 – 1.058)** | **<0.001** | **1.049 (1.023 – 1.076)** | **0.013** | **1.033 (1.007 – 1.060)** | **0.017** | **1.042 (1.007 – 1.077)** |  |  |  |  |
| Age | 0.702 | 1.005 (0.978 – 1.034) |  |  | 0.354 | 0.983 (0.948 – 1.019) |  |  |  |  |  |  |
| Sex | 0.892 | 1.045 (0.552 – 1.981) |  |  | 0.334 | 1.426 (0.694 – 2.928) |  |  |  |  |  |  |
| UICC/BCLC stage | 0.254 | 2.310 (0.548 – 9.737) |  |  | 0.493 | 1.768 (0.346 – 9.024) |  |  |  |  |  |  |
| Tumor entity | 0.515 | 1.055 (0.898 – 1.239) |  |  | **0.063** | **1.198 (0.990 – 1.450)** | **0.004** | **1.334 (1.098 – 1.621)** |  |  |  |  |
| Prior therapy | **0.017** | **2.257 (1.159 – 4.395)** | 0.169 | 1.653 (0.807 – 3.384) | **0.029** | **2.124 (1.081 – 4.173)** | 0.811 | 1.102 (0.499 – 2.434) |  |  |  |  |
| ICI regimen | 0.213 | 0.805 (0.572 – 1.133) |  |  | 0.014 | 0.661 (0.475 – 0.920) |  |  |  |  |  |  |
| ECOG PS | **0.003** | **2.273 (1.328 – 3.892)** | 0.056 | 1.789 (0.986 – 3.247) | **0.001** | **2.642 (1.492 – 4.681)** | **0.005** | **2.804 (1.374 – 5.725)** |  |  |  |  |
| AFP | 0.159 | 1.000 (1.000 – 1.001) |  |  | 0.662 | 1.000 (0.999 – 1.001) |  |  |  |  |  |  |
| ALT | 0.192 | 0.993 (0.983 – 1.003) |  |  | 0.290 | 1.006 (0.995 – 1.017) |  |  |  |  |  |  |
| AST | 0.526 | 0.997 (0.987 – 1.007) |  |  | 0.232 | 1.010 (0.994 – 1.026) |  |  |  |  |  |  |
| Bilirubin | 0.422 | 1.101 (0.870 – 1.394) |  |  | 0.609 | 0.910 (0.634 – 1.307) |  |  |  |  |  |  |
| Creatinine | 0.573 | 1.206 (0.629 – 2.311) |  |  | 0.403 | 0.760 (0.399 – 1.447) |  |  |  |  |  |  |
| LDH | 0.888 | 1.000 (0.997 – 1.003) |  |  | 0.724 | 1.000 (0.997 – 1.002) |  |  |  |  |  |  |
| TPS | 0.268 | 1.007 (0.995 – 1.020) |  |  | 0.317 | 0.993 (0.980 – 1.007) |  |  |  |  |  |  |
| NLR | **0.008** | **1.141 (1.034 – 1.258)** | **0.016** | **1.139 (0.986 – 3.247)** | **0.015** | **1.039 (1.008 – 1.071)** | 0.132 | 1.028 (0.992 – 1.066) |  |  |  |  |

**Supplementary Table 4.** Values for soluble and EV-bound CD27 across different materials (serum/plasma) and dates (March and September 2024)

| **Sample** | **ID** | **sCD27_Plasma_Mar [pg/ml]** | **sCD27_Plasma_Sep [pg/ml]** | **sCD27_Serum_Sep [pg/ml]** | **EVCD27_Serum_Mar [pg/µg]** | **EVCD27_Serum_Sep [pg/µg]** | **EVCD27_Plasma_Sep [pg/µg]** |
| --- | --- | --- | --- | --- | --- | --- | --- |
| P1 | HCCAB22 | 3771.94 | 2527.06 | NA | 1.79 | 1.96 | 1.33 |
| P2 | HCCAB27 | 4402.94 | 5002.56 | 1129.97 | 2.13 | 2.07 | 2 |
| P3 | HCCAB64 | 9079.63 | 12973.11 | 18388.23 | 2.71 | 2.23 | 1.85 |
| P4 | HCCAB68 | 1304.53 | 1508.28 | 1231.02 | 2.17 | 1.97 | 1.82 |
| P5 | HCCAB99 | 493.58 | 788.77 | 285.64 | 2.08 | 2.03 | 1.63 |

## **Supplementary figure legends**

**Supplementary Figure 1.**

Quality assessment of EV isolation from human blood samples. Nanoparticle tracking analysis (Nanosight) with corresponding size distribution and estimated particle concentration (A) and TEM imaging (B) results of a representative HCC patient. (C) Immunolabeling of EVs with EV-markers CD9 and CD63 (C), as well as HCC-related marker Glypican 3 (D).

**Supplementary Figure 2.**

(A) Using a normalized value related to the median of controls for sCD7 of cancer patients (ratio to median controls), no significant differences are observed between sCD27 levels in the training (Aachen ICI) and validation (Hamburg ICI) cohorts. (B-D) Using the same normalized sCD27 value, significant differences between cancers and controls are displayed across all three cohorts. (E-G) ROC curve analysis shows a clear difference between cancer patients and healthy donors across all three cohorts. **p<0.01; ****p<0.0001

**Supplementary Figure 3.**

(A-C) sCD27 concentrations displayed as ratio to median controls according to best response in the ICI-treated *Aachen* (A) *and Hamburg 1* (B) *cohorts*, as well as the non-ICI treated Hamburg 2 cohort (C). *p<0.05

**Supplementary Figure 4.**

(A-C) sCD27 concentrations according to survival status 6 months after therapy initiation in the ICI-treated *Aachen* (A) *and Hamburg 1* (B) *cohorts*, as well as the non-ICI treated Hamburg 2 cohort (C). (D-E) sCD27 concentrations according to immune-related adverse events in the ICI-treated *Aachen* (D) *and Hamburg 1* (E) *cohorts*. **p<0.01

**Supplementary Figure 5.**

Concerning tumor type (A), sex (B) and age (C), no significant differences in sCD27 levels can be seen across all patients from both cohorts (Aachen and Hamburg ICI cohort mixed). (D) No correlation between age and sCD27 levels could be found.

**Supplementary Figure 6.**

Kaplan Meier curves for PFS (A, B, E) and OS (C, D, F) stratified by median sCD27 levels (A, C), as well as ideal baseline cut-off of ratio to median controls CD27 calculated for PFS (B, D-F) in the ICI-treated *Aachen cohort* (A-C) *and Hamburg 1 cohort* (E-F).

**Supplementary Figure 7.**

(A-B) Kaplan Meier curves for PFS (A) and OS (B) stratified by tumor proportion score of PDL-1 (TPS) over 1% in the ICI-treated *Aachen cohort.* (C) TPS according to best response to therapy in the ICI-treated *Aachen cohort* (D-E) Kaplan Meier curves for PFS (A) and OS (B) stratified by a TPS over 1% in the ICI-treated *Hamburg cohort.* (F) TPS according to best response to therapy in the ICI-treated *Hamburg cohort*.**p<0.01

**Supplementary Figure 8.**

(A-B) Kaplan Meier curves for PFS (A) and OS (B) stratified by ideal baseline cut-off of neutrophil lymphocyte ratio (NLR) calculated for PFS in the ICI-treated *Aachen cohort.* (C) NLR according to best response to therapy in the ICI-treated *Aachen cohort* (D-E) Kaplan Meier curves for PFS (A) and OS (B) stratified by the *Aachen* PFS ideal baseline cut-off of NLR in the ICI-treated *Hamburg cohort.* (F) NLR according to best response to therapy in the ICI-treated *Hamburg cohort.*

**Supplementary Figure 9.**

(A-B) sCD27 ratio to median controls in cancer patients and healthy controls are significantly different*: mixed training cohort* (n=77 cancer patients, n=34 controls) (A), *mixed validation cohort* (n=77 cancer patients, n=32 controls) (B). (C-D) sCD27 concentrations displayed as ratio to median controls according to best response in the *mixed training* (C) *and mixed validation* (D) *cohorts*. *p<0.05, **p<0.01, ****p<0.0001

**Supplementary Figure 10.**

Kaplan Meier curves for PFS (A, C) and OS (B, D) stratified by ideal baseline cut-off of ratio to median controls CD27 (calculated for PFS in the *mixed training cohort)* in the *mixed training* (A-B) and *mixed validation cohorts* (C-D).

**Supplementary Figure 11.**

Kaplan Meier curves for PFS (A, D, F, I) and OS (B, E, G, J) stratified by ideal baseline cut-off of ratio to median controls CD27 (calculated for PFS within each cohort*),* as well as box plots depicting differences in objective response according to sCD27 values (C, F, H, K) in the *NSCLC* (A-C), *HCC* (D-F), *melanoma* (F-H) *and other GI tumor cohorts* (I-K) among all patients in the training and validation cohorts. *p<0.05

**Supplementary Figure 12.**

(A-D) Kaplan Meier curves for PFS (A, C) and OS (B, D) stratified by ideal baseline cut-off of serum sCD27 levels throughout therapy calculated for PFS in the ICI-treated *Aachen cohort.*

**Supplementary Figure 13.**

Box plots depicting differences between sCD27 levels according to response at 3 months (A) and 6 months (B) for all patients with available response evaluation and sCD27 levels at all time points. Repeated measures ANOVA showing longitudinal variation of sCD27 levels among different response and survival groups at 3 (B) and 6 months (E). (C) For patients transitioning from PR at 3 months and PD at 6 months (n=2) sCD27 levels show a substantial increase while for patients deepening response (PR at 3 months to CR at 6 months, n=3), levels show a stabilization or even slight decrease. (F) Box plots depicting significant differences in sCD27 levels between patients according to survival status at 6 months. *p<0.05, **p<0.01

**Supplementary Figure 14.**

(A-D) Kaplan Meier curves for PFS (A, C) and OS (B, D) stratified by increasing or decreasing serum sCD27 levels throughout therapy in the ICI-treated *Aachen cohort.*

**Supplementary Figure 15.**

(A-D) Kaplan Meier curves for PFS (A, C) and OS (B, D) stratified by relation of time point specific serum sCD27 levels throughout therapy to the respective ideal cut-off in the ICI-treated *Aachen cohort.*

**Supplementary Figure 16.**

(A) Correlation between soluble CD27 in plasma and CD27 levels on EVs extracted from serum in a subset of the *Hamburg ICI cohort* (n=36). (B-C) Kaplan Meier curves for PFS (B) and OS (C) stratified by increasing or decreasing EV bound CD27 between baseline and the 12-week time point in a subset of the ICI-treated *Hamburg 1 cohort.*
