# Supplementary material for: Soluble and EV-bound CD27 act as antagonistic biomarkers in patients with solid tumors undergoing immunotherapy
Source: J Exp Clin Cancer Res. 2024 Nov 8;43:298. doi: 10.1186/s13046-024-03215-4 (PMC11545160; doi:10.1186/s13046-024-03215-4)
Supplement: Supplementary file 2 — Supplementary Material 2. [file 13046_2024_3215_MOESM2_ESM.pptx]

## Slide 1
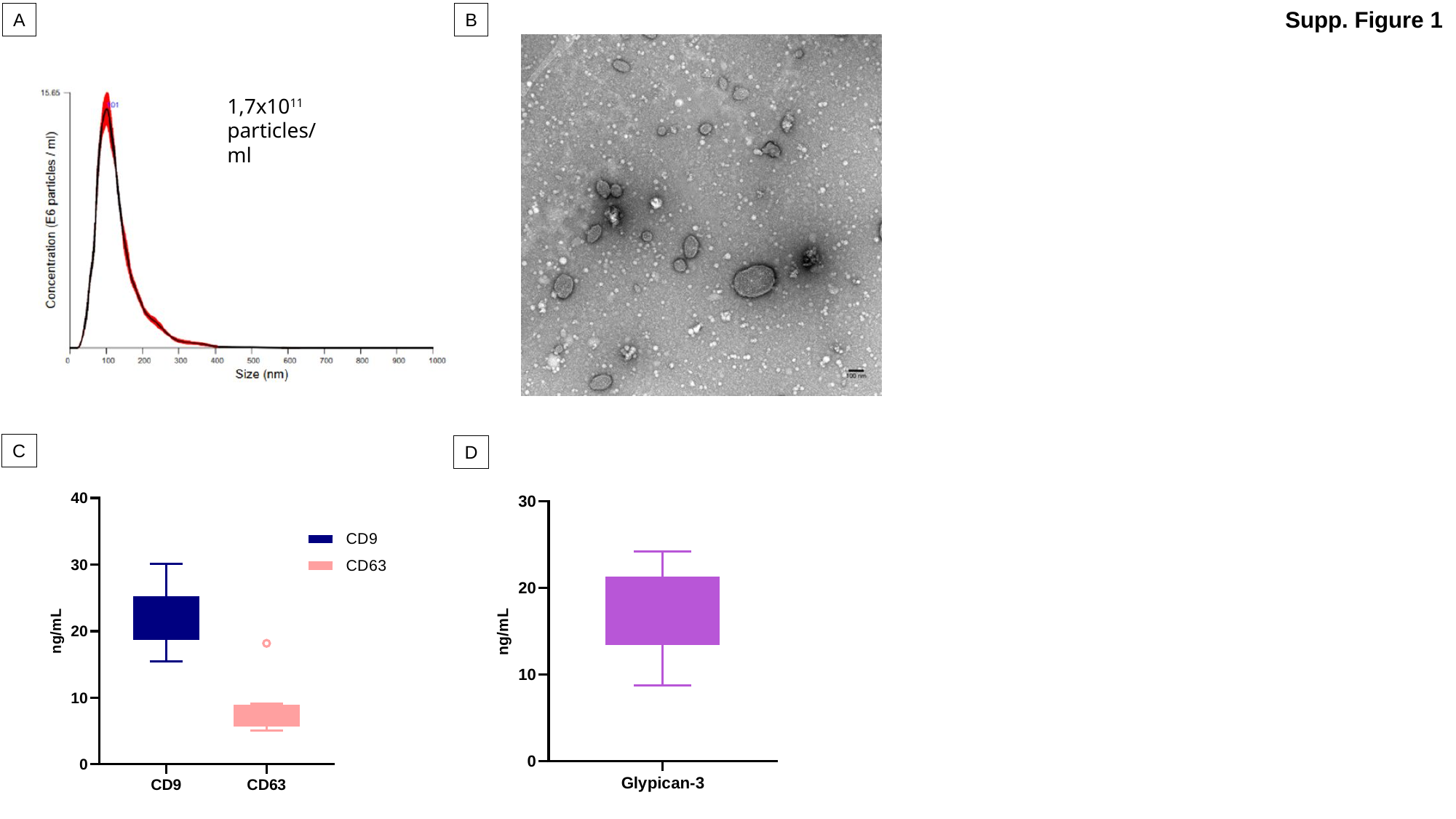

Supp. Figure 1
A
B
1,7x10^11^ particles/ml
C
D

## Slide 2
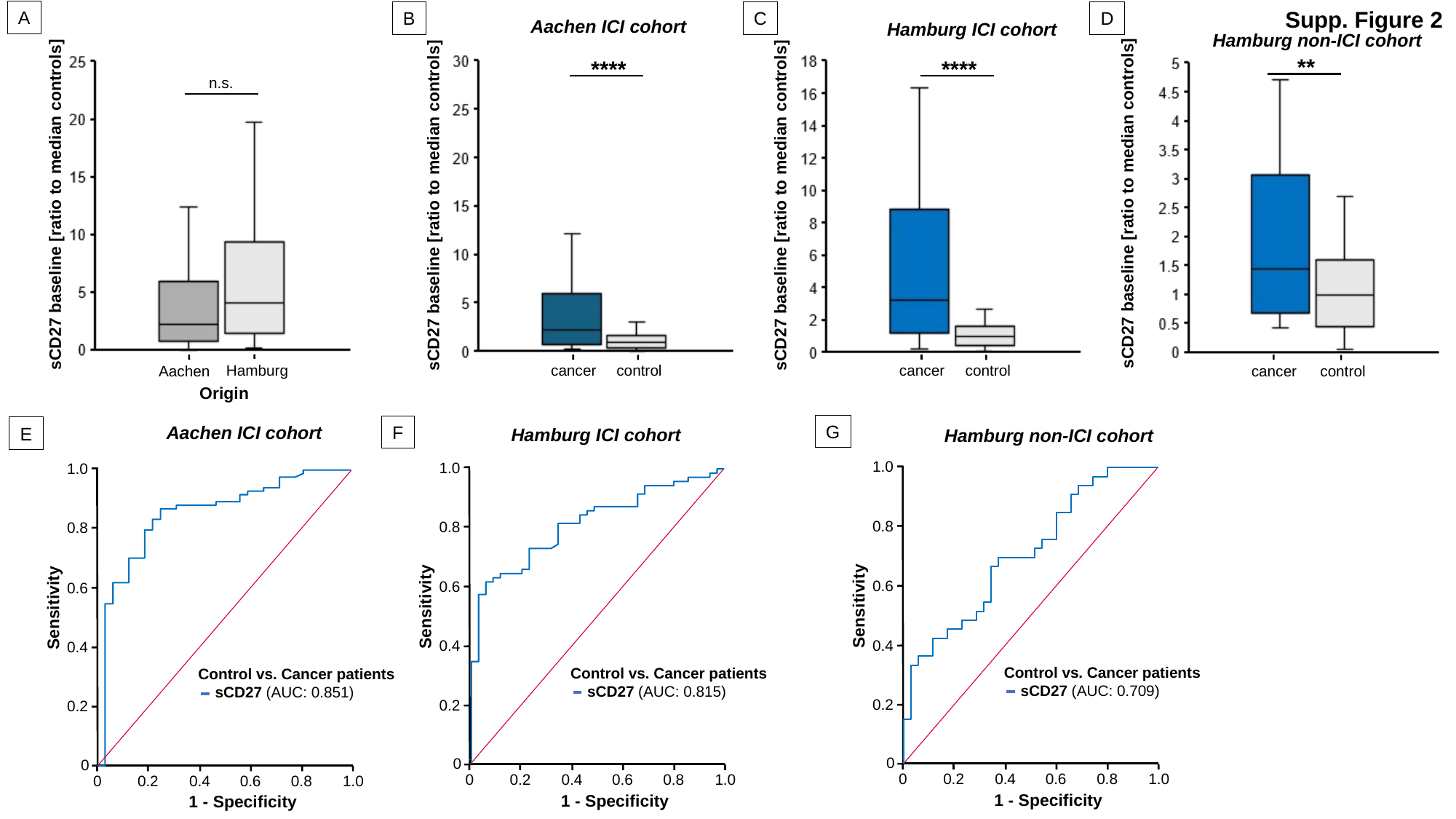

Supp. Figure 2
A
B
C
D
Aachen ICI cohort
Hamburg ICI cohort
Hamburg non-ICI cohort
****
sCD27 baseline [ratio to median controls]
cancer
control
**
****
n.s.
sCD27 baseline [ratio to median controls]
sCD27 baseline [ratio to median controls]
sCD27 baseline [ratio to median controls]
Aachen
Hamburg
cancer
control
cancer
control
Origin
G
Hamburg non-ICI cohort
1.0
0.8
0.6
Sensitivity
0.4
Control vs. Cancer patients
 sCD27 (AUC: 0.709)
0.2
0
0
0.2
0.4
0.6
0.8
1.0
1 - Specificity
F
Hamburg ICI cohort
1.0
0.8
0.6
Sensitivity
0.4
Control vs. Cancer patients
 sCD27 (AUC: 0.815)
0.2
0
0
0.2
0.4
0.6
0.8
1.0
1 - Specificity
E
1.0
0.8
0.6
Sensitivity
0.4
Control vs. Cancer patients
 sCD27 (AUC: 0.851)
0.2
0
0
0.2
0.4
0.6
0.8
1.0
1 - Specificity
Aachen ICI cohort

## Slide 3
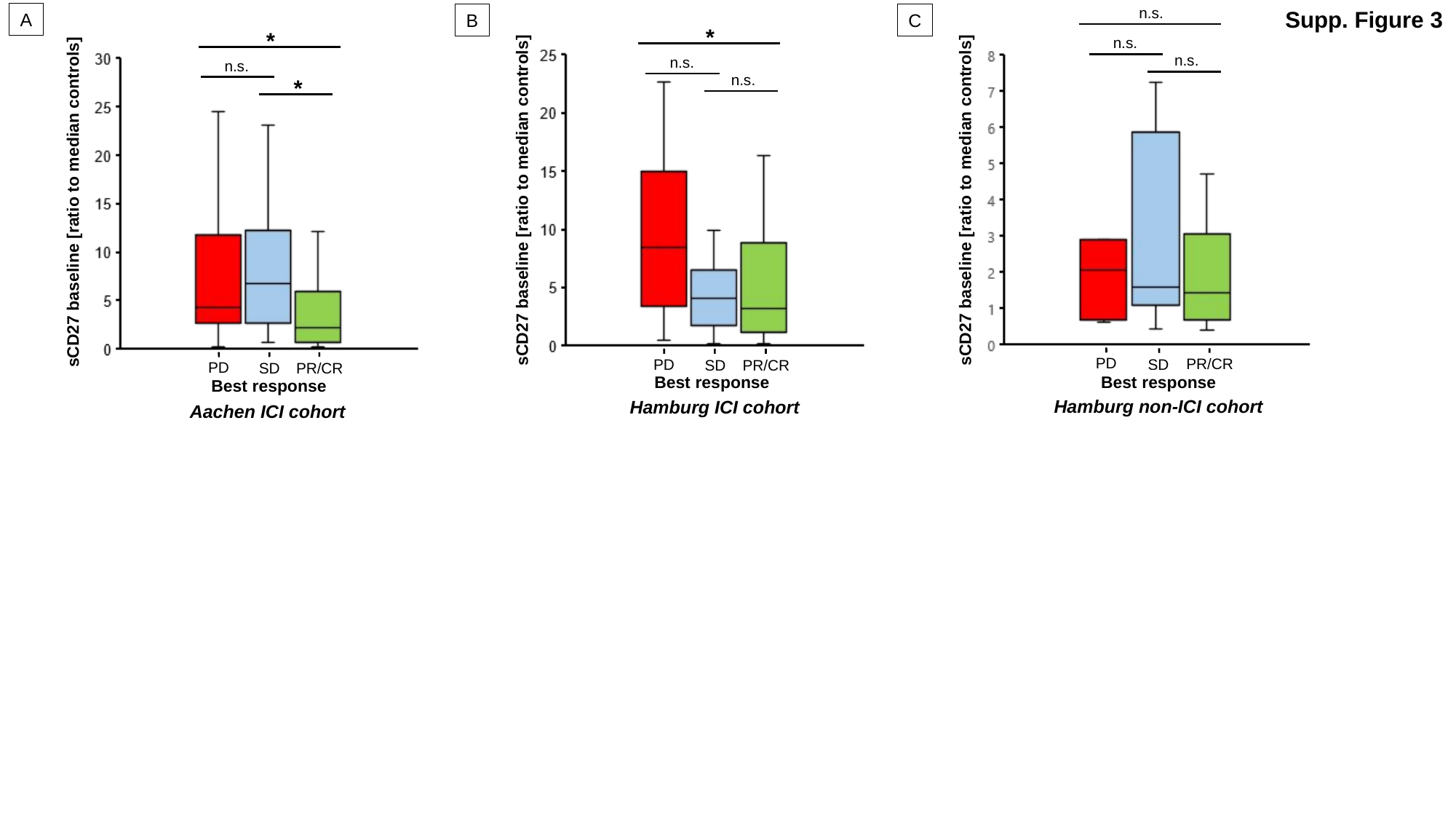

Supp. Figure 3
A
n.s.
B
C
*
*
n.s.
n.s.
n.s.
n.s.
n.s.
*
sCD27 baseline [ratio to median controls]
sCD27 baseline [ratio to median controls]
sCD27 baseline [ratio to median controls]
PD
PR/CR
SD
PD
PR/CR
SD
PD
PR/CR
SD
Best response
Best response
Best response
Hamburg non-ICI cohort
Hamburg ICI cohort
Aachen ICI cohort

## Slide 4
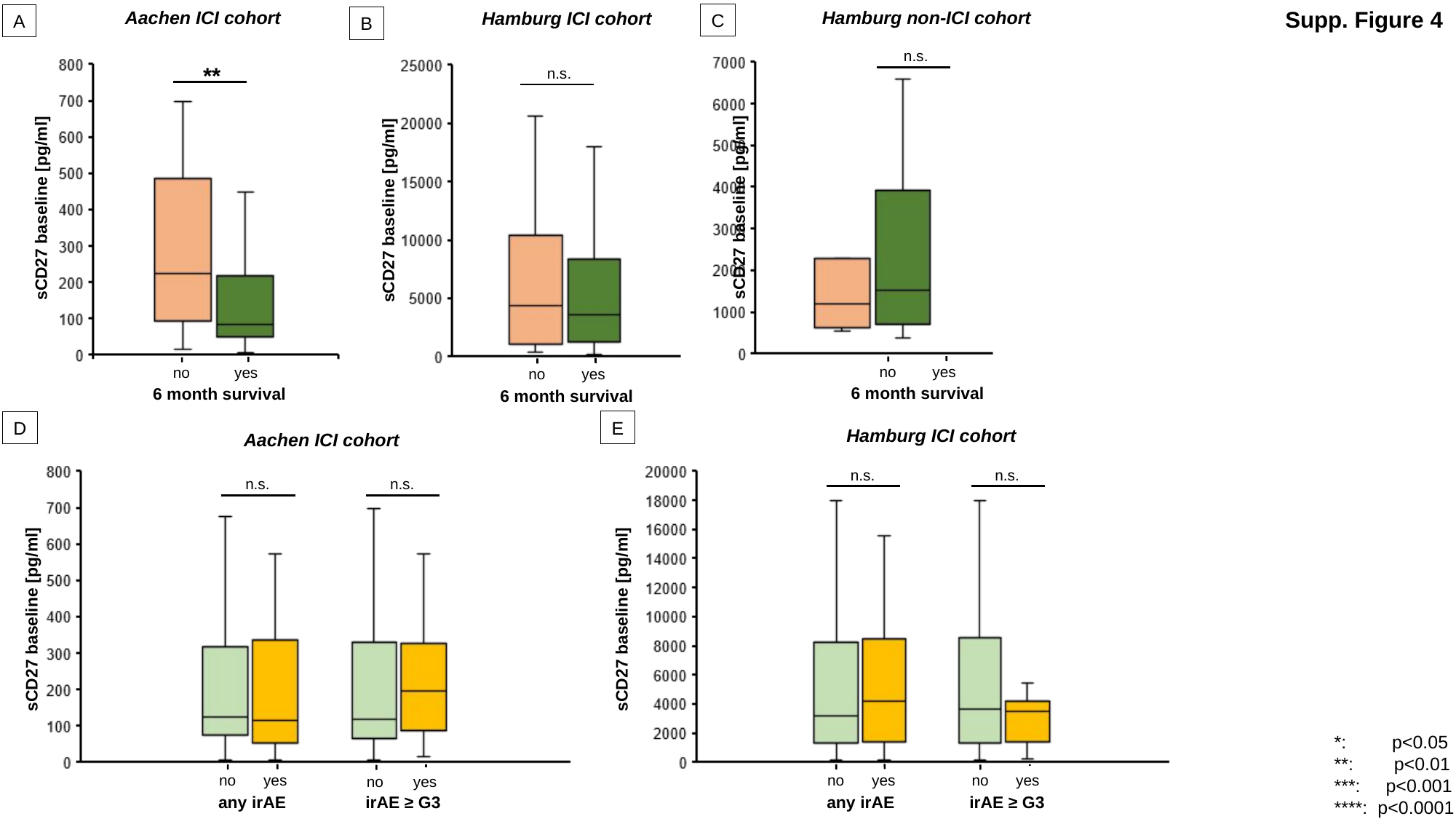

Supp. Figure 4
C
n.s.
sCD27 baseline [pg/ml]
no
yes
6 month survival
A
Hamburg non-ICI cohort
Aachen ICI cohort
Hamburg ICI cohort
B
**
n.s.
sCD27 baseline [pg/ml]
sCD27 baseline [pg/ml]
no
yes
no
yes
6 month survival
6 month survival
E
D
Hamburg ICI cohort
Aachen ICI cohort
n.s.
n.s.
n.s.
n.s.
sCD27 baseline [pg/ml]
sCD27 baseline [pg/ml]
*: p<0.05
**: p<0.01
***: p<0.001
****: p<0.0001
no
no
no
yes
no
yes
yes
yes
any irAE
any irAE
 irAE ≥ G3
 irAE ≥ G3

## Slide 5
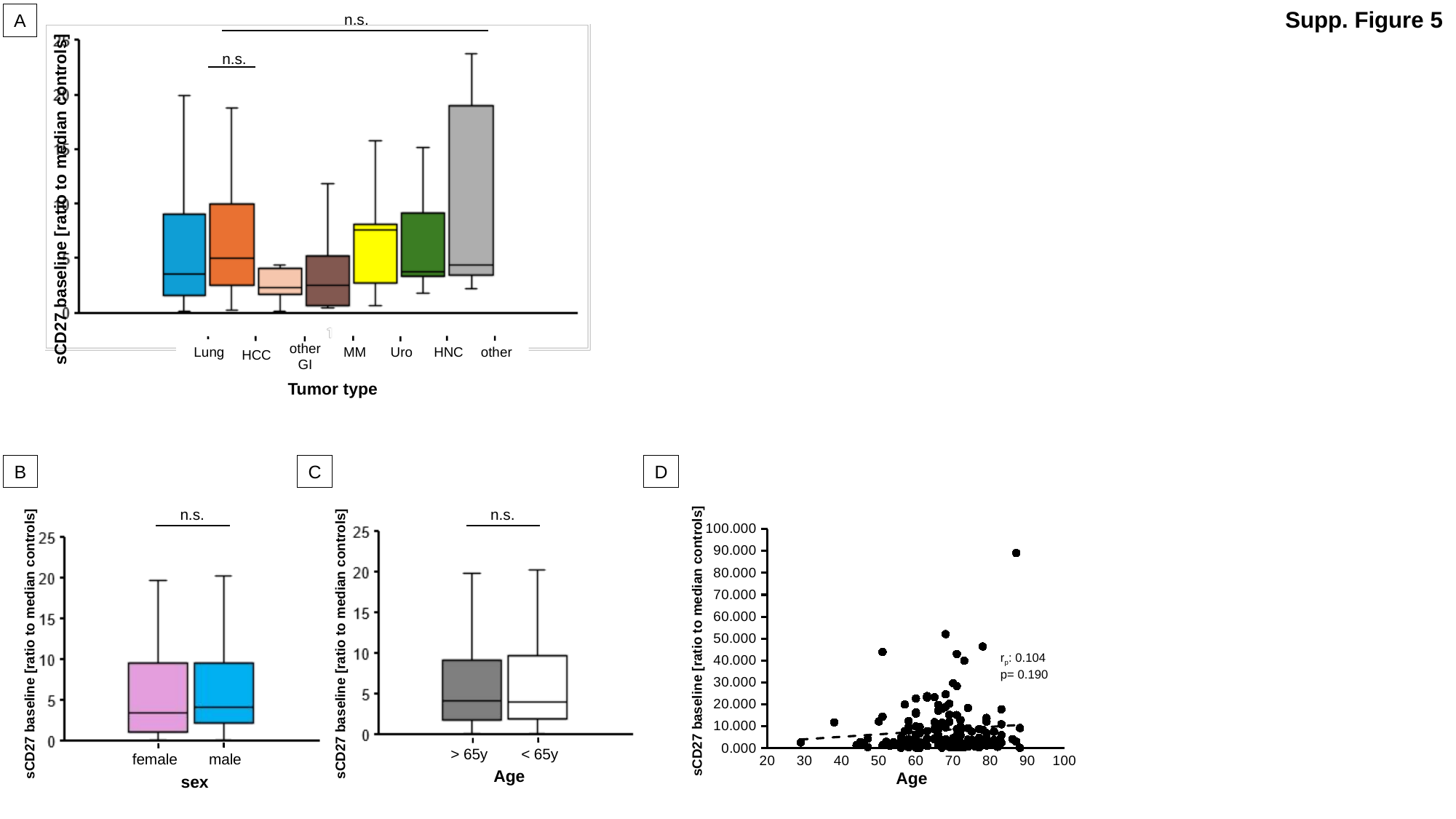

Supp. Figure 5
A
n.s.
n.s.
sCD27 baseline [ratio to median controls]
Lung
MM
Uro
HNC
other
HCC
other GI
Tumor type
D
C
B
n.s.
n.s.
### Chart
| Category | |
|---|---|sCD27 baseline [ratio to median controls]
sCD27 baseline [ratio to median controls]
sCD27 baseline [ratio to median controls]
rp: 0.104
p= 0.190
> 65y
< 65y
female
male
Age
Age
sex

## Slide 6
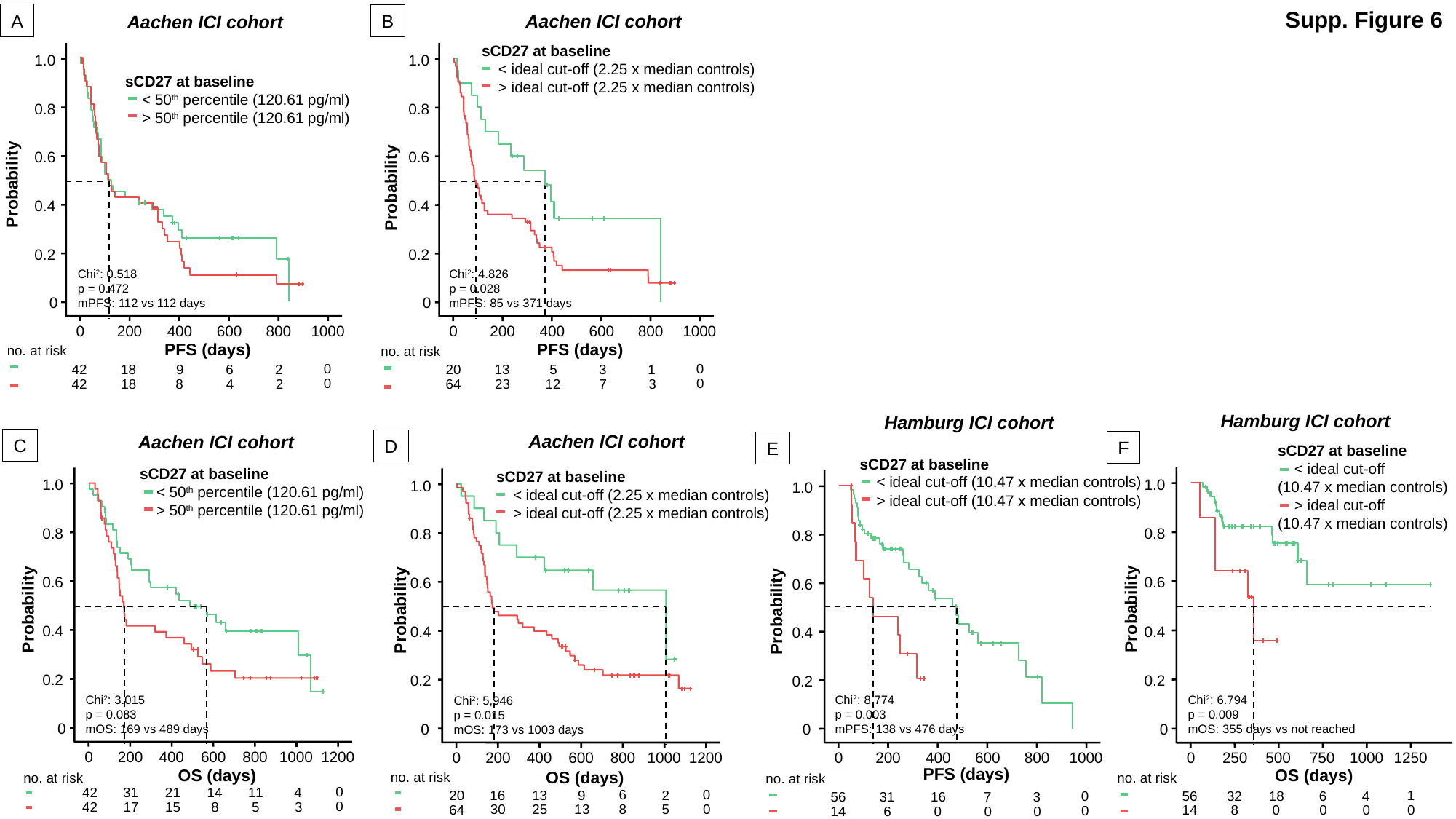

Supp. Figure 6
A
B
Aachen ICI cohort
Aachen ICI cohort
sCD27 at baseline
 < ideal cut-off (2.25 x median controls)
 > ideal cut-off (2.25 x median controls)
1.0
sCD27 at baseline
 < 50th percentile (120.61 pg/ml)
 > 50th percentile (120.61 pg/ml)
0.8
0.6
Probability
0.4
0.2
Chi2: 0.518
p = 0.472
mPFS: 112 vs 112 days
0
0
200
400
600
800
1000
PFS (days)
no. at risk
0
18
9
6
2
42
0
18
8
4
2
42
1.0
0.8
0.6
Probability
0.4
0.2
Chi2: 4.826
p = 0.028
mPFS: 85 vs 371 days
0
0
200
400
600
800
1000
PFS (days)
no. at risk
0
13
5
3
1
20
0
23
12
7
3
64
Hamburg ICI cohort
Hamburg ICI cohort
C
D
F
Aachen ICI cohort
Aachen ICI cohort
E
sCD27 at baseline
 < ideal cut-off (10.47 x median controls)
 > ideal cut-off (10.47 x median controls)
1.0
0.8
0.6
Probability
0.4
0.2
0
0
200
400
600
800
1000
PFS (days)
Chi2: 8.774
p = 0.003
mPFS: 138 vs 476 days
no. at risk
0
31
16
7
3
56
0
6
0
0
0
14
sCD27 at baseline
 < ideal cut-off
(10.47 x median controls)
 > ideal cut-off
(10.47 x median controls)
sCD27 at baseline
 < 50th percentile (120.61 pg/ml)
 > 50th percentile (120.61 pg/ml)
sCD27 at baseline
 < ideal cut-off (2.25 x median controls)
 > ideal cut-off (2.25 x median controls)
1.0
0.8
0.6
0.4
0.2
0
0
250
500
750
1000
1250
OS (days)
1.0
1.0
0.8
0.8
0.6
0.6
Probability
Probability
Probability
0.4
0.4
0.2
0.2
Chi2: 3.015
p = 0.083
mOS: 169 vs 489 days
Chi2: 6.794
p = 0.009
mOS: 355 days vs not reached
Chi2: 5,946
p = 0.015
mOS: 173 vs 1003 days
0
0
1000
0
800
200
400
600
1200
0
800
1000
200
400
600
1200
OS (days)
OS (days)
no. at risk
0
6
16
13
9
2
20
0
8
30
25
13
5
64
no. at risk
1
32
18
6
4
56
0
8
0
0
0
14
no. at risk
0
11
31
21
14
4
42
0
5
17
15
8
3
42

## Slide 7
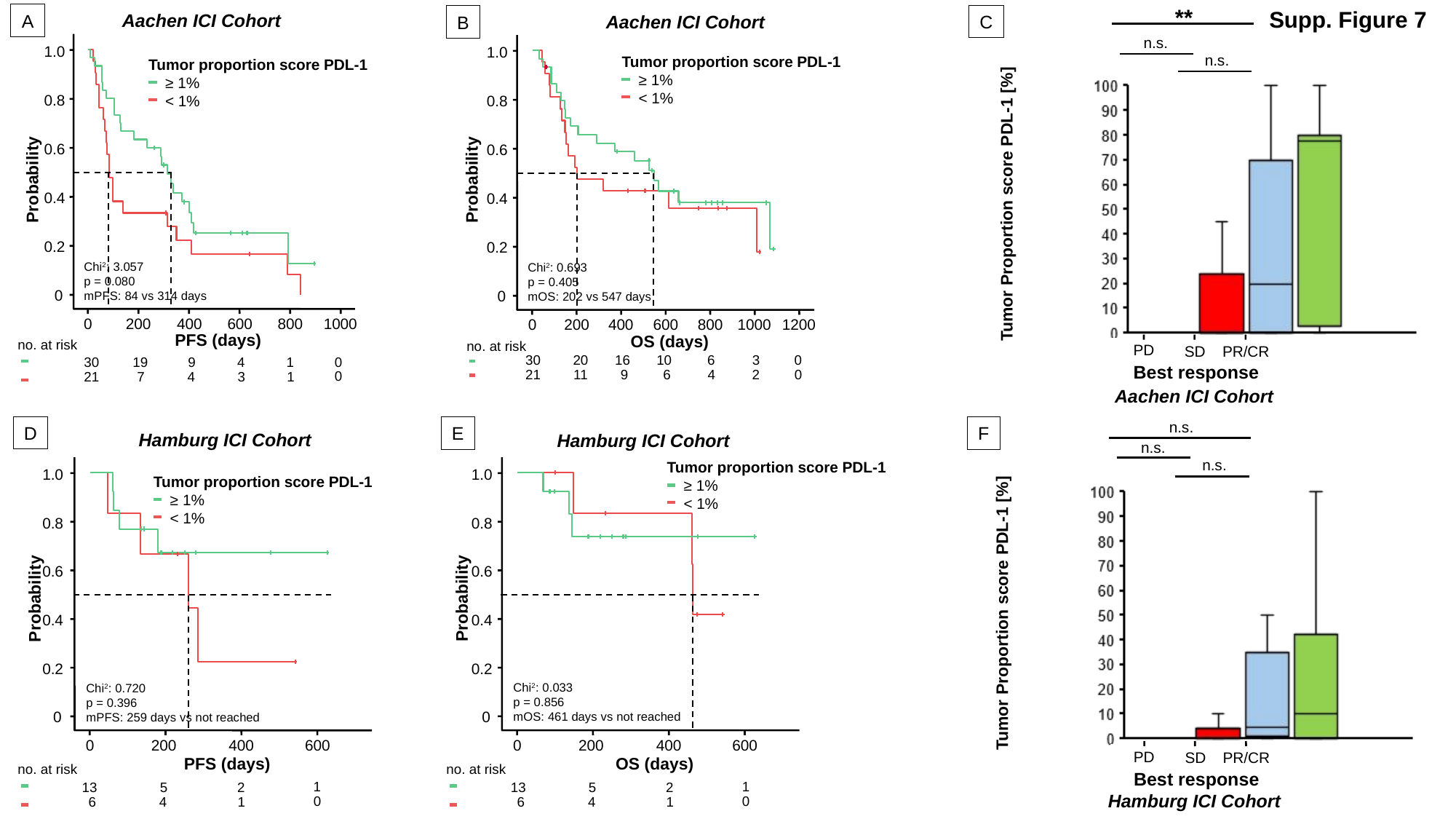

Supp. Figure 7
Aachen ICI Cohort
Aachen ICI Cohort
B
1.0
0.8
0.6
Probability
0.4
0.2
Chi2: 0.693
p = 0.405
mOS: 202 vs 547 days
0
0
800
1000
200
400
600
1200
OS (days)
Tumor proportion score PDL-1
 ≥ 1%
 < 1%
no. at risk
0
6
20
16
10
3
30
4
11
9
6
2
0
21
**
C
n.s.
n.s.
Tumor Proportion score PDL-1 [%]
PD
PR/CR
SD
Best response
Aachen ICI Cohort
A
1.0
Tumor proportion score PDL-1
 ≥ 1%
 < 1%
0.8
0.6
Probability
0.4
0.2
Chi2: 3.057
p = 0.080
mPFS: 84 vs 314 days
0
0
200
400
600
800
1000
PFS (days)
no. at risk
0
19
9
4
1
30
0
7
4
3
1
21
D
Hamburg ICI Cohort
1.0
0.8
0.6
Probability
0.4
0.2
Chi2: 0.720
p = 0.396
mPFS: 259 days vs not reached
0
0
200
400
600
PFS (days)
no. at risk
1
5
2
13
0
4
1
6
F
n.s.
n.s.
n.s.
Tumor Proportion score PDL-1 [%]
PD
PR/CR
SD
Best response
Hamburg ICI Cohort
E
Hamburg ICI Cohort
1.0
0.8
0.6
Probability
0.4
0.2
Chi2: 0.033
p = 0.856
mOS: 461 days vs not reached
0
0
200
400
600
OS (days)
no. at risk
1
5
2
13
0
4
1
6
Tumor proportion score PDL-1
 ≥ 1%
 < 1%
Tumor proportion score PDL-1
 ≥ 1%
 < 1%

## Slide 8
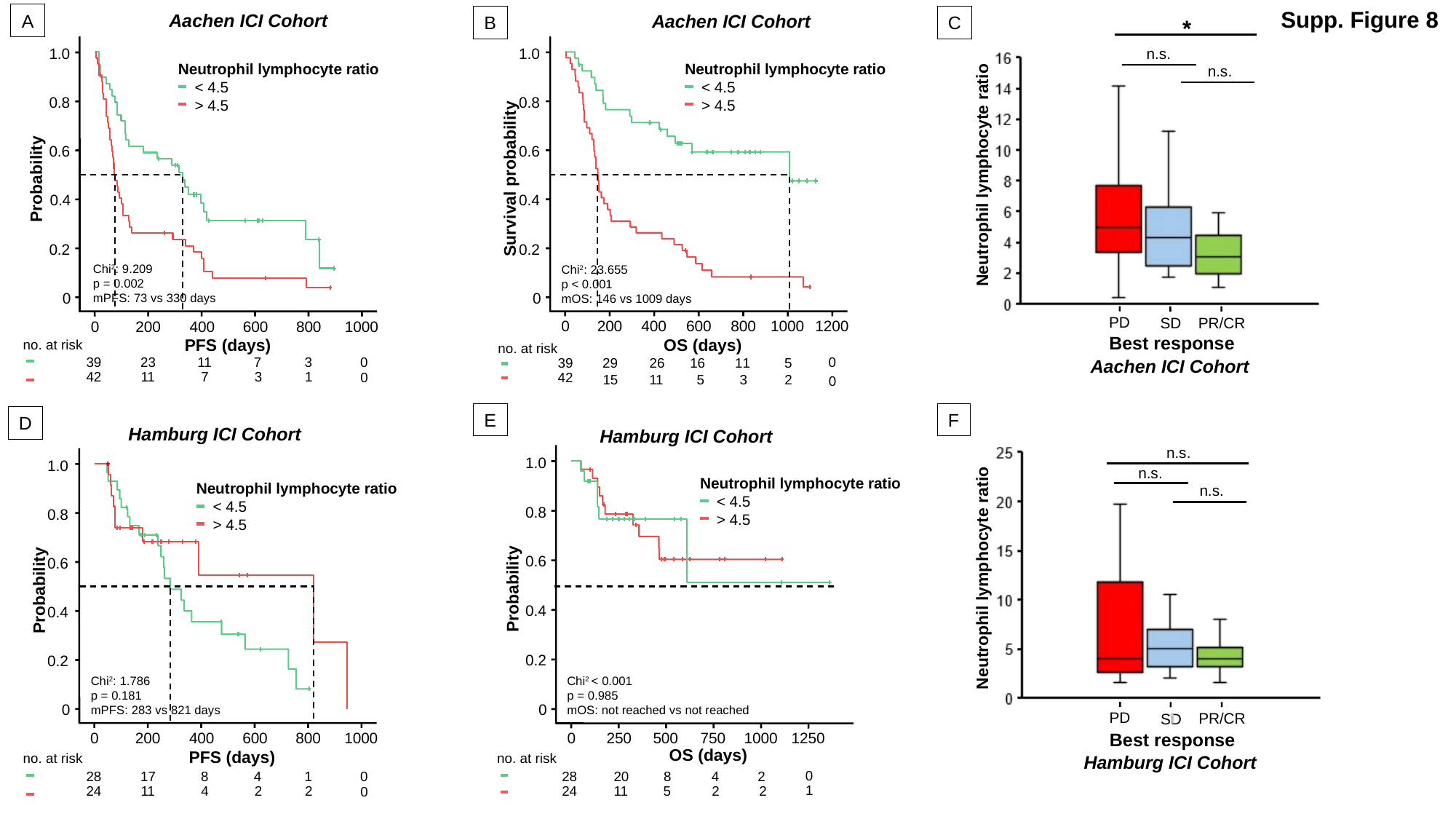

Supp. Figure 8
Aachen ICI Cohort
Aachen ICI Cohort
A
B
C
*
1.0
0.8
0.6
Survival probability
0.4
0.2
0
0
800
1000
200
400
600
1200
OS (days)
1.0
0.8
0.6
Probability
0.4
0.2
Chi2: 9.209
p = 0.002
mPFS: 73 vs 330 days
0
0
200
400
600
800
1000
PFS (days)
n.s.
Neutrophil lymphocyte ratio
 < 4.5
 > 4.5
Neutrophil lymphocyte ratio
 < 4.5
 > 4.5
n.s.
Neutrophil lymphocyte ratio
Chi2: 23.655
p < 0.001
mOS: 146 vs 1009 days
PD
PR/CR
SD
Best response
no. at risk
no. at risk
Aachen ICI Cohort
0
0
23
11
7
3
39
11
29
26
16
5
39
11
7
3
1
42
42
0
3
15
11
5
2
0
E
F
D
Hamburg ICI Cohort
Hamburg ICI Cohort
n.s.
1.0
0.8
0.6
Probability
0.4
0.2
Chi2 < 0.001
p = 0.985
mOS: not reached vs not reached
0
0
250
500
750
1000
1250
OS (days)
1.0
0.8
0.6
Probability
0.4
0.2
Chi2: 1.786
p = 0.181
mPFS: 283 vs 821 days
0
0
200
400
600
800
1000
PFS (days)
n.s.
Neutrophil lymphocyte ratio
 < 4.5
 > 4.5
Neutrophil lymphocyte ratio
 < 4.5
 > 4.5
n.s.
Neutrophil lymphocyte ratio
PD
PR/CR
SD
Best response
Hamburg ICI Cohort
no. at risk
no. at risk
0
0
20
8
4
2
28
17
8
4
1
28
1
11
5
2
2
24
11
4
2
2
24
0

## Slide 9
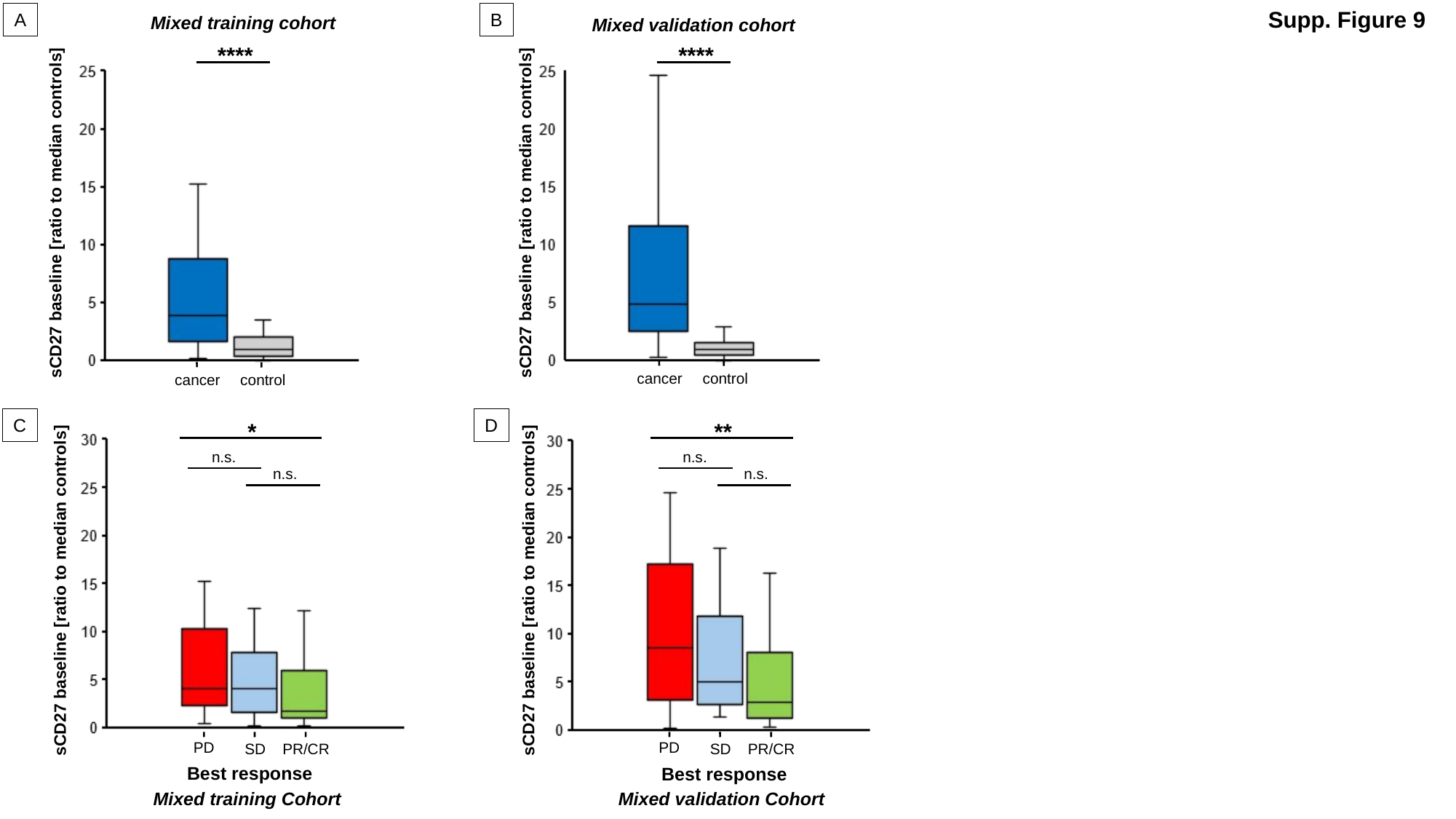

Supp. Figure 9
Mixed training cohort
A
B
Mixed validation cohort
****
****
sCD27 baseline [ratio to median controls]
sCD27 baseline [ratio to median controls]
cancer
control
cancer
control
C
D
*
**
n.s.
n.s.
n.s.
n.s.
sCD27 baseline [ratio to median controls]
sCD27 baseline [ratio to median controls]
PD
PD
PR/CR
PR/CR
SD
SD
Best response
Best response
Mixed training Cohort
Mixed validation Cohort

## Slide 10
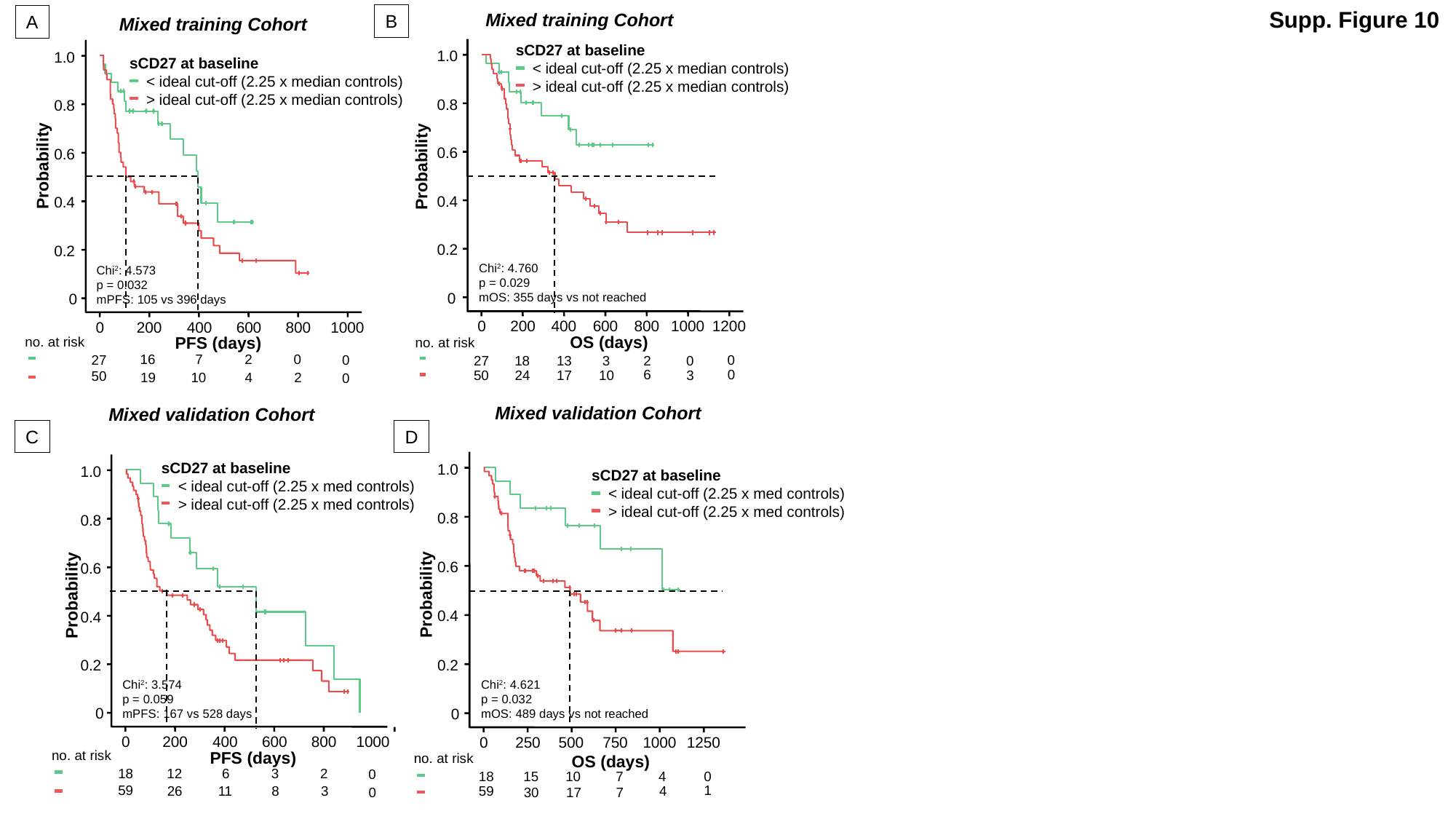

Mixed training Cohort
Supp. Figure 10
Mixed training Cohort
B
A
sCD27 at baseline
 < ideal cut-off (2.25 x median controls)
 > ideal cut-off (2.25 x median controls)
1.0
0.8
0.6
Probability
0.4
0.2
Chi2: 4.760
p = 0.029
mOS: 355 days vs not reached
0
0
800
1000
200
400
600
1200
OS (days)
1.0
0.8
0.6
Probability
0.4
0.2
Chi2: 4.573
p = 0.032
mPFS: 105 vs 396 days
0
0
200
400
600
800
1000
PFS (days)
sCD27 at baseline
 < ideal cut-off (2.25 x median controls)
 > ideal cut-off (2.25 x median controls)
no. at risk
16
7
2
0
27
0
50
19
10
4
2
0
no. at risk
0
2
18
13
3
0
27
0
6
24
17
10
3
50
Mixed validation Cohort
Mixed validation Cohort
C
D
sCD27 at baseline
 < ideal cut-off (2.25 x med controls)
 > ideal cut-off (2.25 x med controls)
1.0
sCD27 at baseline
 < ideal cut-off (2.25 x med controls)
 > ideal cut-off (2.25 x med controls)
1.0
0.8
0.8
0.6
0.6
Probability
Probability
0.4
0.4
0.2
0.2
Chi2: 4.621
p = 0.032
mOS: 489 days vs not reached
Chi2: 3.574
p = 0.059
mPFS: 167 vs 528 days
0
0
0
200
400
600
800
1000
0
250
500
750
1000
1250
no. at risk
12
6
3
2
18
0
59
26
11
8
3
0
PFS (days)
no. at risk
0
15
10
7
4
18
1
4
59
30
17
7
OS (days)

## Slide 11
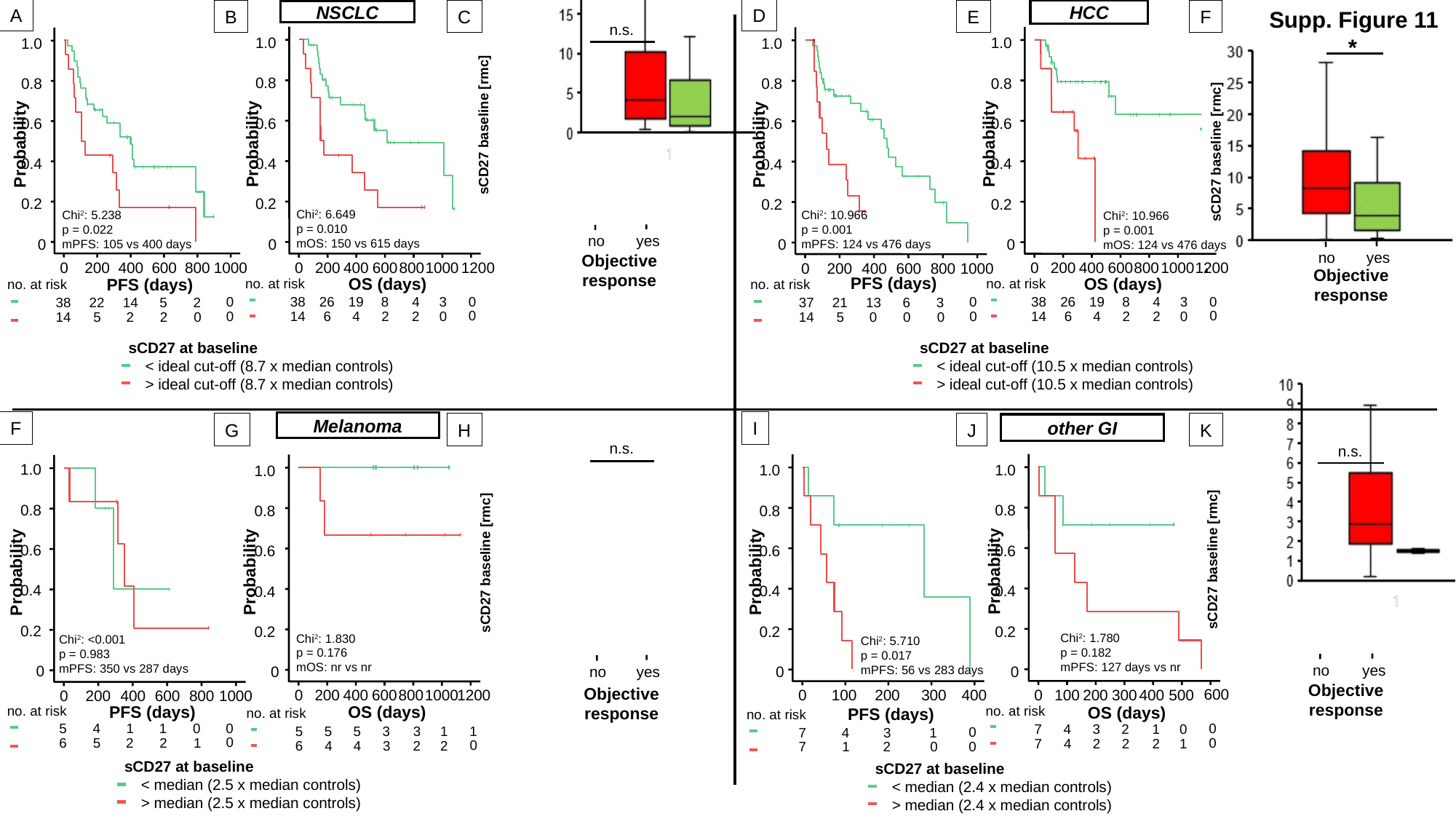

A
D
HCC
Supp. Figure 11
C
B
F
E
NSCLC
sCD27 baseline [rmc]
Objective response
n.s.
1.0
0.8
0.6
Probability
0.4
0.2
Chi2: 5.238
p = 0.022
mPFS: 105 vs 400 days
0
0
200
400
600
800
1000
PFS (days)
no. at risk
0
22
14
5
2
38
0
5
2
2
0
14
*
1.0
1.0
1.0
0.8
0.8
0.8
0.6
0.6
0.6
Probability
Probability
Probability
sCD27 baseline [rmc]
0.4
0.4
0.4
0.2
0.2
0.2
Chi2: 6.649
p = 0.010
mOS: 150 vs 615 days
Chi2: 10.966
p = 0.001
mPFS: 124 vs 476 days
Chi2: 10.966
p = 0.001
mOS: 124 vs 476 days
no
yes
0
0
0
no
yes
0
800
1000
200
400
600
1200
0
800
1000
200
400
600
1200
0
200
400
600
800
1000
PFS (days)
OS (days)
no. at risk
0
4
26
19
8
3
38
0
2
6
4
2
0
14
OS (days)
Objective response
no. at risk
no. at risk
0
4
26
19
8
3
38
0
21
13
6
3
37
0
0
2
6
4
2
0
14
5
0
0
0
14
sCD27 at baseline
 < ideal cut-off (8.7 x median controls)
 > ideal cut-off (8.7 x median controls)
sCD27 at baseline
 < ideal cut-off (10.5 x median controls)
 > ideal cut-off (10.5 x median controls)
F
I
Melanoma
H
G
K
J
other GI
n.s.
sCD27 baseline [rmc]
no
yes
Objective response
n.s.
sCD27 baseline [rmc]
no
yes
Objective response
1.0
0.8
0.6
Probability
0.4
0.2
0
600
0
400
500
100
200
300
OS (days)
no. at risk
0
1
4
3
2
0
7
0
2
4
2
2
1
7
1.0
0.8
0.6
Probability
0.4
0.2
0
0
800
1000
200
400
600
1200
OS (days)
1.0
1.0
0.8
0.8
0.6
0.6
Probability
Probability
0.4
0.4
0.2
0.2
Chi2: 1.780
p = 0.182
mPFS: 127 days vs nr
Chi2: 1.830
p = 0.176
mOS: nr vs nr
Chi2: <0.001
p = 0.983
mPFS: 350 vs 287 days
Chi2: 5.710
p = 0.017
mPFS: 56 vs 283 days
0
0
0
100
200
300
400
0
200
400
600
800
1000
PFS (days)
no. at risk
0
4
1
1
0
5
0
5
2
2
1
6
PFS (days)
no. at risk
1
3
5
5
3
1
5
0
2
4
4
3
2
6
no. at risk
0
4
3
1
7
0
1
2
0
7
sCD27 at baseline
 < median (2.5 x median controls)
 > median (2.5 x median controls)
sCD27 at baseline
 < median (2.4 x median controls)
 > median (2.4 x median controls)

## Slide 12
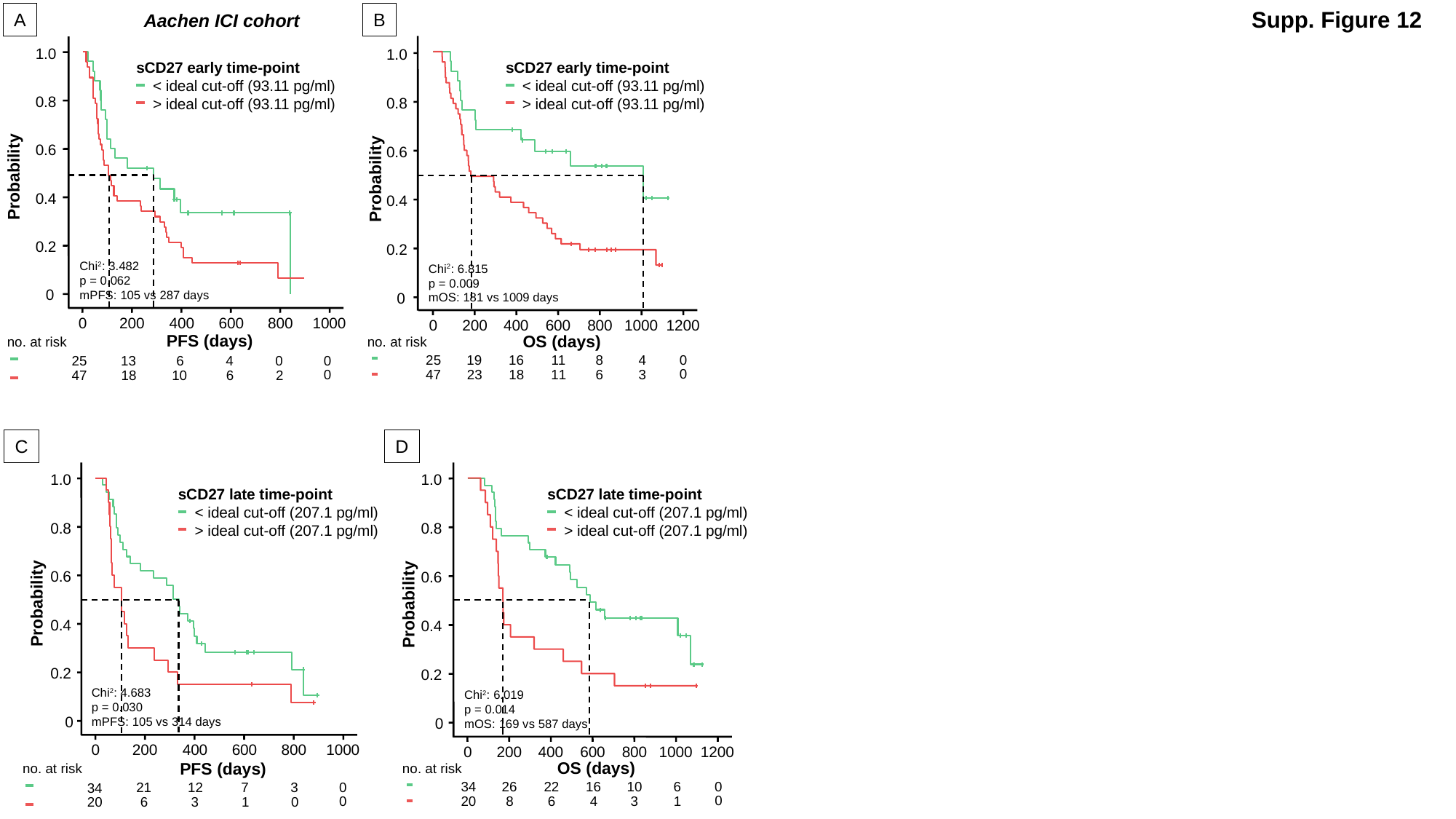

Supp. Figure 12
A
B
Aachen ICI cohort
1.0
1.0
sCD27 early time-point
 < ideal cut-off (93.11 pg/ml)
 > ideal cut-off (93.11 pg/ml)
sCD27 early time-point
 < ideal cut-off (93.11 pg/ml)
 > ideal cut-off (93.11 pg/ml)
0.8
0.8
0.6
0.6
Probability
Probability
0.4
0.4
0.2
0.2
Chi2: 3.482
p = 0.062
mPFS: 105 vs 287 days
Chi2: 6.815
p = 0.009
mOS: 181 vs 1009 days
0
0
0
200
400
600
800
1000
0
800
1000
200
400
600
1200
PFS (days)
OS (days)
no. at risk
0
13
6
4
0
25
0
18
10
6
2
47
no. at risk
0
8
19
16
11
4
25
0
6
23
18
11
3
47
C
D
1.0
0.8
0.6
Probability
0.4
0.2
0
0
200
400
600
800
1000
PFS (days)
1.0
sCD27 late time-point
 < ideal cut-off (207.1 pg/ml)
 > ideal cut-off (207.1 pg/ml)
sCD27 late time-point
 < ideal cut-off (207.1 pg/ml)
 > ideal cut-off (207.1 pg/ml)
0.8
0.6
Probability
0.4
0.2
Chi2: 4.683
p = 0.030
mPFS: 105 vs 314 days
Chi2: 6.019
p = 0.014
mOS: 169 vs 587 days
0
0
800
1000
200
400
600
1200
OS (days)
no. at risk
0
10
26
22
16
6
34
0
3
8
6
4
1
20
no. at risk
0
21
12
7
3
34
0
6
3
1
0
20

## Slide 13
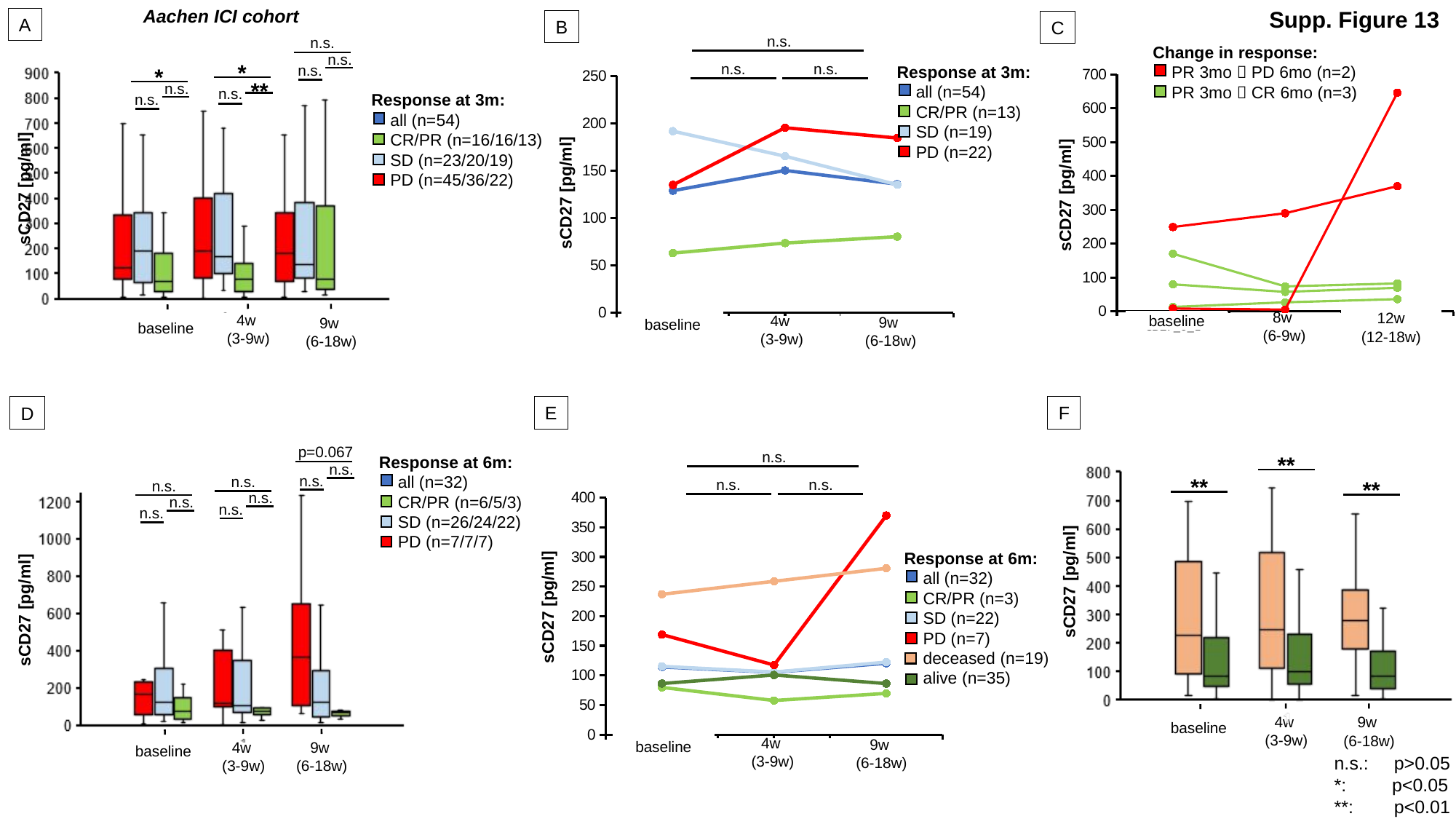

Supp. Figure 13
Aachen ICI cohort
A
n.s.
n.s.
*
n.s.
*
**
n.s.
n.s.
Response at 3m:
 all (n=54)
 CR/PR (n=16/16/13)
 SD (n=23/20/19)
 PD (n=45/36/22)
n.s.
sCD27 [pg/ml]
4w
(3-9w)
baseline
9w
(6-18w)
B
n.s.
n.s.
n.s.
### Chart
| Category | | | | |
|---|---|---|---|---|
| CD27_base | 128.99 | 63.1 | 191.76 | 135.05 |
| CD27_t1 | 150.33 | 73.7 | 165.35 | 195.39 |
| CD27_t2 | 135.9 | 80.46 | 135.28 | 184.51 |sCD27 [pg/ml]
baseline
9w
(6-18w)
4w
(3-9w)
C
### Chart
| Category | TA | BR | NM | KR | TR |
|---|---|---|---|---|---|
| CD27_0_1 | 170.06 | 79.68 | 13.08 | 8.2 | 249.13 |
| CD27_23 | 73.7 | 57.52 | 26.65 | 5.1 | 289.83 |
| CD27_456 | 82.35 | 69.58 | 36.04 | 645.93 | 369.99 |sCD27 [pg/ml]
12w
(12-18w)
baseline
8w
(6-9w)
Change in response:
 PR 3mo  PD 6mo (n=2)
 PR 3mo  CR 6mo (n=3)
Response at 3m:
 all (n=54)
 CR/PR (n=13)
 SD (n=19)
 PD (n=22)
E
F
D
p=0.067
Response at 6m:
 all (n=32)
 CR/PR (n=6/5/3)
 SD (n=26/24/22)
 PD (n=7/7/7)
n.s.
**
n.s.
n.s.
n.s.
**
n.s.
n.s.
**
n.s.
### Chart
| Category | | | | | | |
|---|---|---|---|---|---|---|n.s.
n.s.
n.s.
n.s.
Response at 6m:
 all (n=32)
 CR/PR (n=3)
 SD (n=22)
 PD (n=7)
 deceased (n=19)
 alive (n=35)
sCD27 [pg/ml]
sCD27 [pg/ml]
sCD27 [pg/ml]
baseline
4w
(3-9w)
9w
(6-18w)
baseline
9w
(6-18w)
baseline
4w
(3-9w)
4w
(3-9w)
9w
(6-18w)
n.s.: p>0.05
*: p<0.05
**: p<0.01

## Slide 14
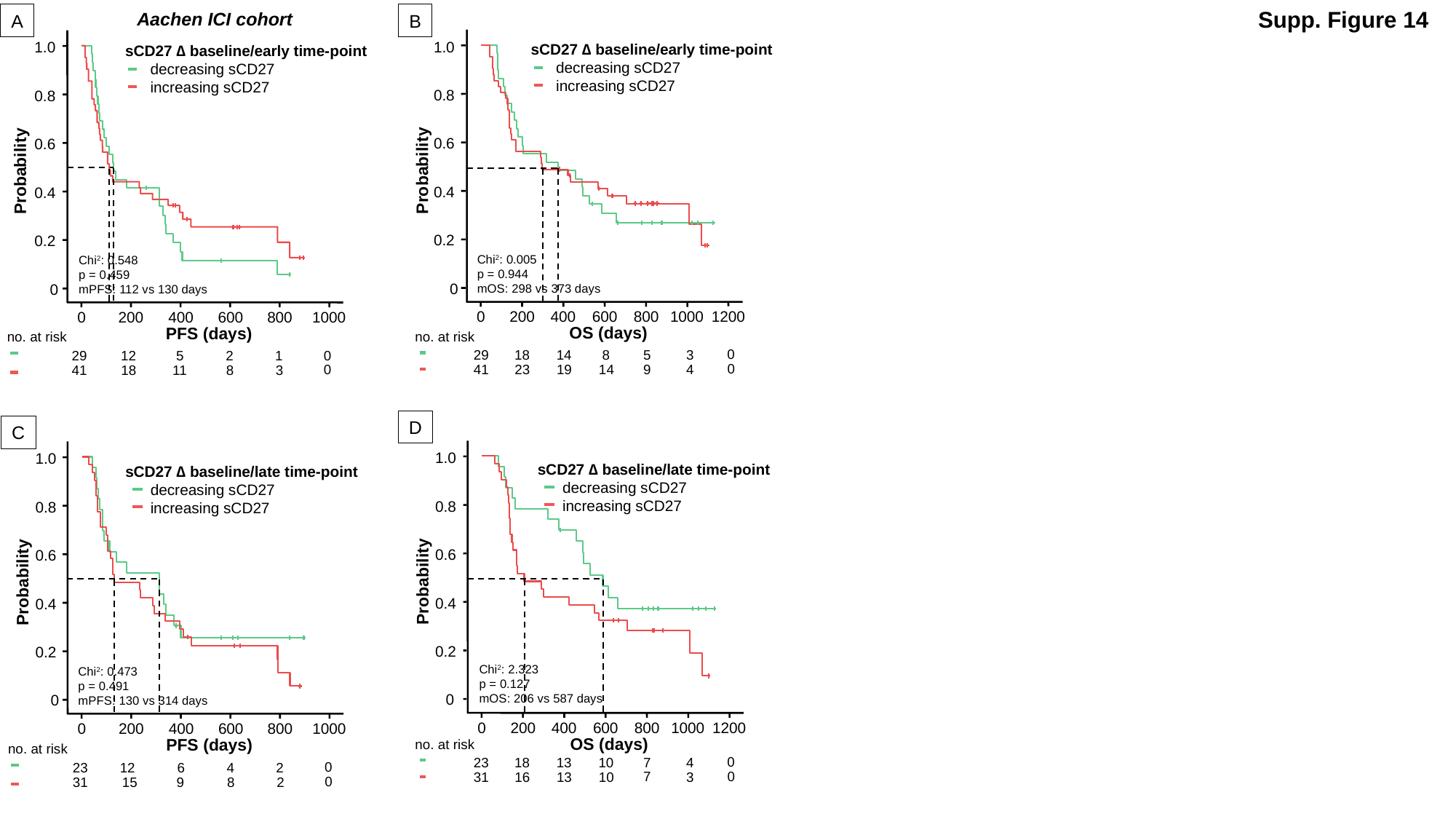

Supp. Figure 14
A
B
sCD27 ∆ baseline/early time-point
 decreasing sCD27
 increasing sCD27
1.0
0.8
0.6
Probability
0.4
0.2
Chi2: 0.005
p = 0.944
mOS: 298 vs 373 days
0
0
800
1000
200
400
600
1200
OS (days)
no. at risk
0
5
18
14
8
3
29
0
9
23
19
14
4
41
Aachen ICI cohort
sCD27 ∆ baseline/early time-point
 decreasing sCD27
 increasing sCD27
1.0
0.8
0.6
Probability
0.4
0.2
Chi2: 0.548
p = 0.459
mPFS: 112 vs 130 days
0
0
200
400
600
800
1000
PFS (days)
no. at risk
0
12
5
2
1
29
0
18
11
8
3
41
D
1.0
0.8
0.6
Probability
0.4
0.2
0
0
800
1000
200
400
600
1200
OS (days)
sCD27 ∆ baseline/late time-point
 decreasing sCD27
 increasing sCD27
Chi2: 2.323
p = 0.127
mOS: 206 vs 587 days
no. at risk
0
7
18
13
10
4
23
0
7
16
13
10
3
31
C
1.0
0.8
0.6
Probability
0.4
0.2
0
0
200
400
600
800
1000
PFS (days)
sCD27 ∆ baseline/late time-point
 decreasing sCD27
 increasing sCD27
Chi2: 0.473
p = 0.491
mPFS: 130 vs 314 days
no. at risk
0
12
6
4
2
23
0
15
9
8
2
31

## Slide 15
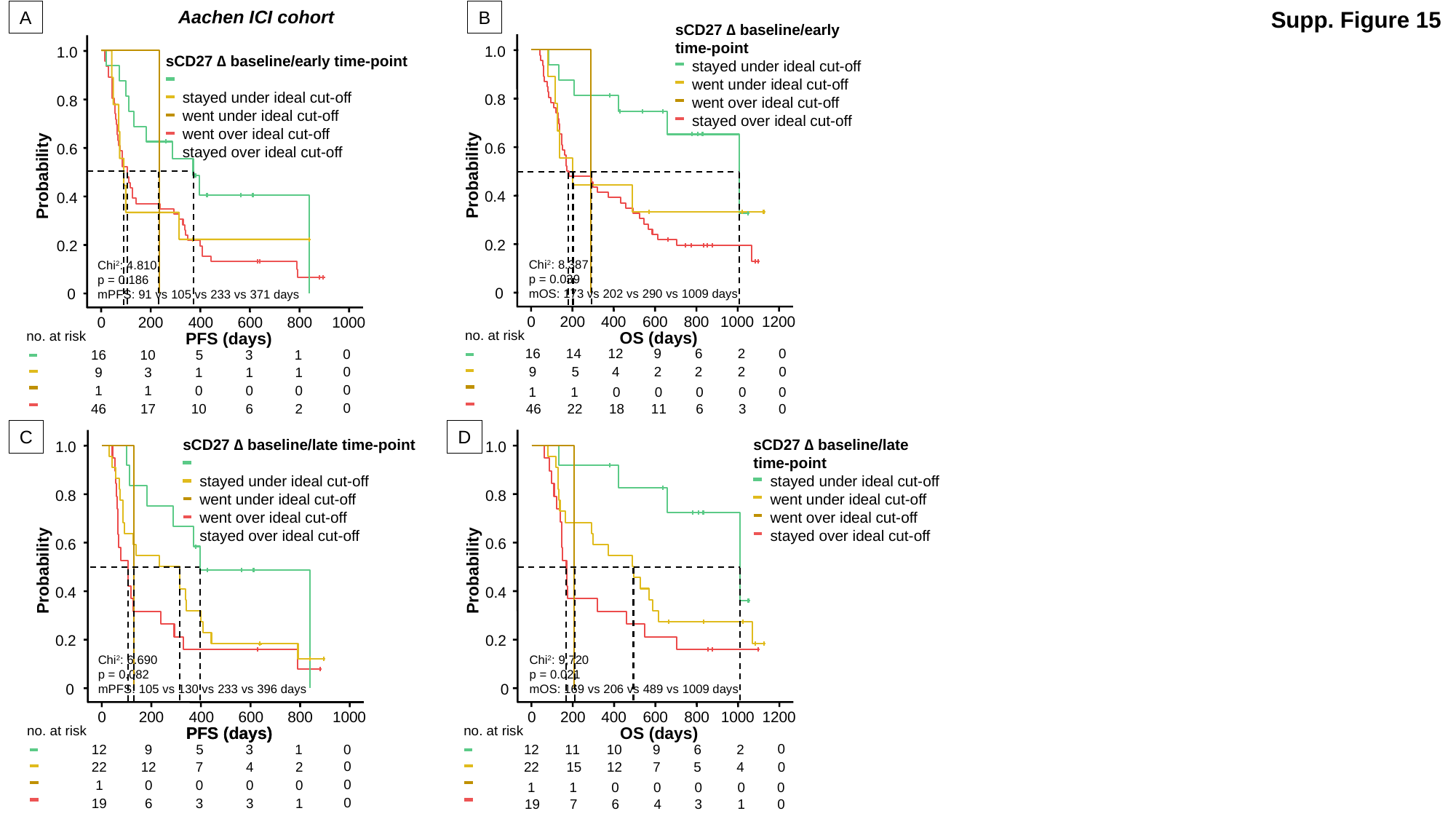

Supp. Figure 15
A
B
sCD27 ∆ baseline/early
time-point
 stayed under ideal cut-off
 went under ideal cut-off
 went over ideal cut-off
 stayed over ideal cut-off
1.0
0.8
0.6
Probability
0.4
0.2
Chi2: 8.387
p = 0.039
mOS: 173 vs 202 vs 290 vs 1009 days
0
0
800
1000
200
400
600
1200
no. at risk
0
6
14
12
9
2
16
9
5
4
2
2
2
0
1
1
0
0
0
0
0
46
22
18
11
6
3
0
OS (days)
Aachen ICI cohort
1.0
sCD27 ∆ baseline/early time-point
 stayed under ideal cut-off
 went under ideal cut-off
 went over ideal cut-off
 stayed over ideal cut-off
0.8
0.6
Probability
0.4
0.2
Chi2: 4.810
p = 0.186
mPFS: 91 vs 105 vs 233 vs 371 days
0
0
200
400
600
800
1000
no. at risk
PFS (days)
0
10
5
3
1
16
0
3
1
1
1
9
0
1
0
0
0
1
0
17
10
6
2
46
D
C
sCD27 ∆ baseline/late
time-point
 stayed under ideal cut-off
 went under ideal cut-off
 went over ideal cut-off
 stayed over ideal cut-off
sCD27 ∆ baseline/late time-point
 stayed under ideal cut-off
 went under ideal cut-off
 went over ideal cut-off
 stayed over ideal cut-off
1.0
0.8
0.6
Probability
0.4
0.2
0
0
200
400
600
800
1000
PFS (days)
1.0
0.8
0.6
Probability
0.4
0.2
Chi2: 9,720
p = 0.021
mOS: 169 vs 206 vs 489 vs 1009 days
Chi2: 6.690
p = 0.082
mPFS: 105 vs 130 vs 233 vs 396 days
0
0
800
1000
200
400
600
1200
no. at risk
PFS (days)
0
9
5
3
1
12
0
12
7
4
2
22
0
0
0
0
0
1
0
6
3
3
1
19
no. at risk
OS (days)
0
6
11
10
9
2
12
22
15
12
7
5
4
0
1
1
0
0
0
0
0
19
7
6
4
3
1
0

## Slide 16
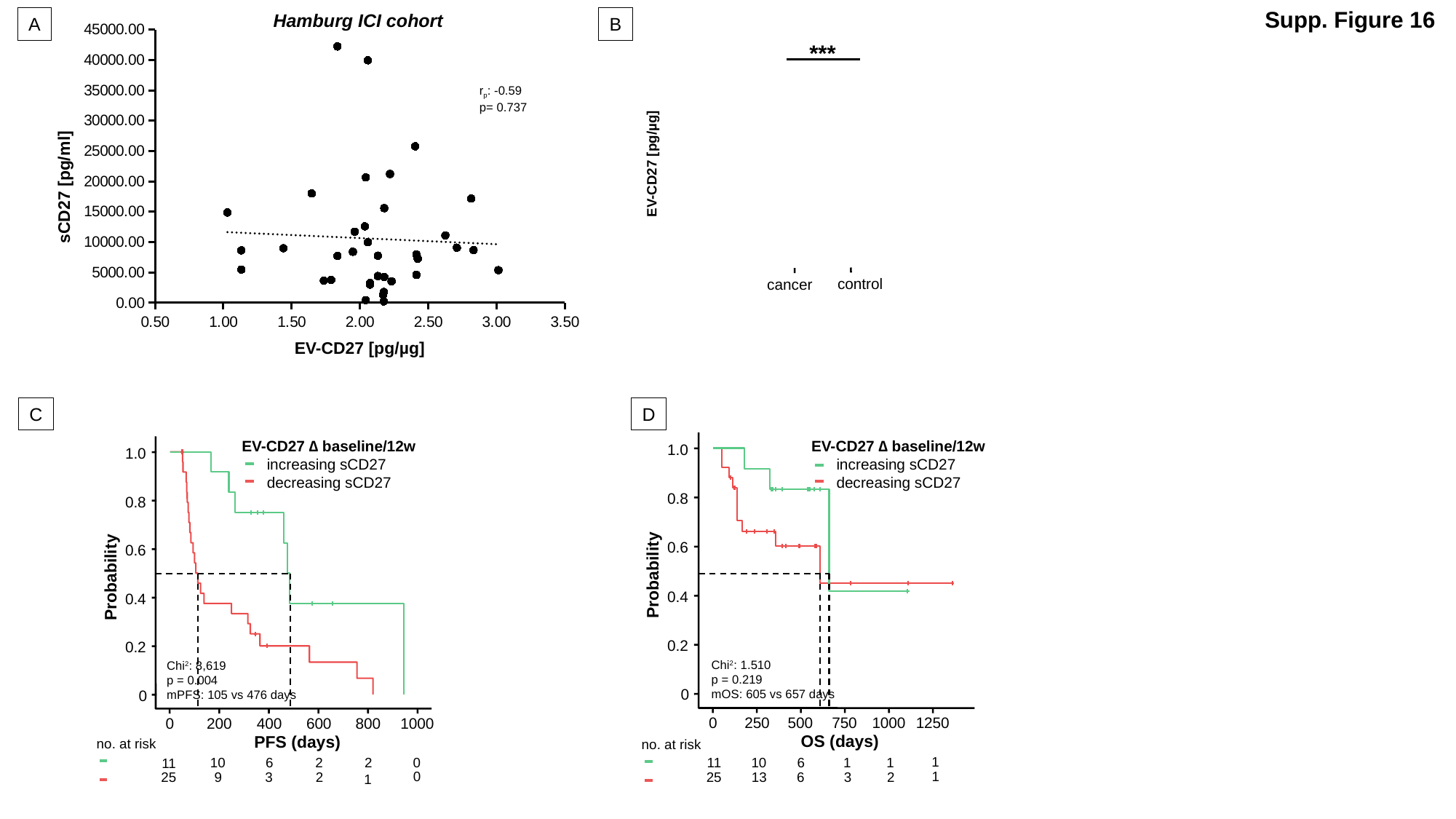

Supp. Figure 16
Hamburg ICI cohort
A
### Chart:
| Category | |
|---|---|rp: -0.59
p= 0.737
sCD27 [pg/ml]
EV-CD27 [pg/µg]
B
***
EV-CD27 [pg/µg]
cancer
control
C
D
EV-CD27 ∆ baseline/12w
 increasing sCD27
 decreasing sCD27
1.0
0.8
0.6
Probability
0.4
0.2
0
0
200
400
600
800
1000
PFS (days)
Chi2: 8,619
p = 0.004
mPFS: 105 vs 476 days
EV-CD27 ∆ baseline/12w
 increasing sCD27
 decreasing sCD27
1.0
0.8
0.6
Probability
0.4
0.2
0
0
250
500
750
1000
1250
OS (days)
Chi2: 1.510
p = 0.219
mOS: 605 vs 657 days
no. at risk
0
10
6
2
2
11
0
9
3
2
25
no. at risk
1
10
6
1
1
11
1
13
6
3
2
25
1
